# Bioinformatics and Deep Learning Approach to Discover Food-Derived Active Ingredients for Alzheimer’s Disease Therapy

**DOI:** 10.3390/foods14010127

**Published:** 2025-01-04

**Authors:** Junyu Zhou, Chen Li, Yong Kwan Kim, Sunmin Park

**Affiliations:** 1 Institute of Advanced Clinical Medicine, Peking University, Beijing 100191, China; zjy888zjy888@gmail.com; 2 Department of Bioconvergence, Hoseo University, Asan 31499, Republic of Korea; lic77732@gmail.com; 3 Department of Information and Communication Engineering, Hoseo University, Asan 31499, Republic of Korea; ykkim@hoseo.edu; 4 Department of Food and Nutrition, Obesity/Diabetes Research Center, Hoseo University, Asan 31499, Republic of Korea

**Keywords:** deep neural analysis, neurogenerative disease, Alzheimer’s disease, flavonoids, inflammation

## Abstract

Alzheimer’s disease (AD) prevention is a critical challenge for aging societies, necessitating the exploration of food ingredients and whole foods as potential therapeutic agents. This study aimed to identify natural compounds (NCs) with therapeutic potential in AD using an innovative bioinformatics-integrated deep neural analysis approach, combining computational predictions with molecular docking and in vitro experiments for comprehensive evaluation. We employed the bioinformatics-integrated deep neural analysis of NCs for Disease Discovery (BioDeepNat) application in the data collected from chemical databases. Random forest regression models were utilized to predict the IC_50_ (pIC_50_) values of ligands interacting with AD-related target proteins, including acetylcholinesterase (*AChE*), amyloid precursor protein (*APP*), beta-secretase 1 (*BACE1*), microtubule-associated protein tau (*MAPT*), presenilin-1 (*PSEN1*), tumor necrosis factor (*TNF*)*-α*, and valosin-containing protein (*VCP*). Their activities were then validated through a molecular docking analysis using Autodock Vina. Predictions by the deep neural analysis identified 166 NCs with potential effects on AD across seven proteins, demonstrating outstanding recall performance. The top five food sources of these predicted compounds were black walnut, safflower, ginger, fig, corn, and pepper. Statistical clustering methodologies segregated the NCs into six well-defined groups, each characterized by convergent structural and chemical signatures. The systematic examination of structure–activity relationships uncovered differential molecular patterns among clusters, illuminating the sophisticated correlation between molecular properties and biological activity. Notably, NCs with high activity, such as astragalin, dihydromyricetin, and coumarin, and medium activity, such as luteolin, showed promising effects in improving cell survival and reducing lipid peroxidation and *TNF-α* expression levels in PC12 cells treated with lipopolysaccharide. In conclusion, our findings demonstrate the efficacy of combining bioinformatics with deep neural networks to expedite the discovery of previously unidentified food-derived active ingredients (NCs) for AD intervention.

## 1. Introduction

Alzheimer’s disease (AD) is a progressive neurodegenerative disorder characterized by cognitive decline, memory loss, and behavioral changes. It is the most common cause of dementia among older adults, affecting millions of elderly people worldwide [1]. The etiology of AD is complex and multifactorial. It is not yet fully understood. However, several genetic and environmental factors have been implicated in its development [1]. Mutations in the amyloid-β precursor protein (*APP*), presenilin (*PSEN*)*-1*, and *PSEN2* genes have been associated with early-onset familial AD, and the apolipoprotein E (*APOE*) ε4 allele is a primary risk factor for late-onset sporadic AD [2,3]. Other genes, such as the triggering receptor expressed on myeloid cells 2 (*TREM2*), sialic acid-binding immunoglobulin-type of lectins (*SIGLEC-3*, *CD33*), and sortilin-related receptor 1 (*SORL1*), have also been linked to AD susceptibility [3]. These genetic factors contribute to pathological processes in AD, including amyloid-β peptide (Aβ) production, tau hyperphosphorylation, and neuroinflammation.

The advent of deep learning techniques has revolutionized medical research, particularly in the context of Alzheimer’s disease (AD). Recent advances demonstrate the utility of multimodal deep learning approaches, which integrate diverse datasets, such as neuroimaging, clinical parameters, and cognitive assessments, to enhance the accuracy of AD diagnosis and progression tracking [4]. These models have been successfully applied to analyze brain imaging modalities like MRI and PET scans, providing the early detection of AD and valuable insights into disease progression [5]. Beyond diagnostics, deep learning has also been instrumental in facilitating the identification of novel therapeutic targets and natural compounds through the integration of bioinformatics with chemical and molecular databases. This convergence of computational power and biological knowledge enables the systematic exploration of structure–activity relationships and the prediction of ligand–protein interactions. Such advances hold a promise for uncovering new therapeutic avenues and optimizing drug discovery processes, particularly in the context of food-derived bioactive compounds,

Natural compounds (NCs) have emerged as promising candidates for preventing and treating AD due to their diverse biological activities and low toxicity. Compounds derived from plants, herbs, and dietary sources have been proven to possess various biological activities relevant to AD, such as antioxidant, anti-inflammatory, and anti-amyloidogenic effects [4]. For example, vinpocetine and ferulic acid have been reported to inhibit Aβ aggregation and reduce neuroinflammation in AD animal models [5]. Similarly, resveratrol and epigallocatechin-3-gallate (EGCG) have been shown to attenuate neuroinflammation and oxidative stress and improve cognitive function in animal studies [6]. However, identifying and validating NCs for AD therapy remains challenging due to the vast number of potential candidates and the complexity of their mechanisms of action.

Traditional approaches, such as in vitro and in vivo screening, are time-consuming and resource-intensive and often have low success rates. In recent years, bioinformatics and deep learning techniques have emerged as powerful tools for drug discovery and repurposing [7]. These approaches leverage large-scale biological and chemical data to predict potential drug-multi-target disease interactions and identify novel therapeutic candidates [8]. By integrating information from various sources, such as gene expression profiles, protein–protein interactions, and chemical databases, bioinformatics-based methods can prioritize compounds with the desired properties and guide experimental validation.

This study aimed to identify and evaluate NCs with therapeutic potential for AD by integrating bioinformatics, deep neural analysis, and in vitro validation. While NCs have been studied for their roles in various diseases, including neurodegenerative disorders, our work uniquely focuses on their structure–activity relationships (SARs), molecular docking, and biological activities specifically in the context of AD. To uncover novel therapeutic applications, we clustered NCs based on their biological activity profiles and selected candidates from diverse structural groups, such as anthocyanins, flavonoids, and other polyphenols, thereby minimizing redundancy with previous studies. This innovative methodology not only identifies promising NCs but also accelerates drug discovery for AD by targeting the specific pathways and molecular mechanisms involved in the disease. Furthermore, our approach highlights the synergistic use of computational techniques, such as deep neural analysis and molecular docking, combined with in vitro validation to comprehensively assess the therapeutic potential of NCs. These efforts are expected to deepen our understanding of NC-target interactions in AD and contribute to the development of effective, disease-modifying therapeutic strategies.

## 2. Materials and Methods

### 2.1. Selection of Target Proteins and Structure Prediction

To identify the molecular targets for AD, we screened for the genes associated with AD risk and AD pathogenesis in the National Center for Biotechnology Information (NCBI, https://www.ncbi.nlm.nih.gov/; 23 August 2023) and GeneCards^®^ (https://www.genecards.org/; 11 October 2023) websites. Genes with higher relevance scores are more likely to be associated with specific functions or pathways related to AD. Three-dimensional chemical structures of the AD-related target proteins (encoded by the genes identified above) were predicted using the AlphaFold Protein Structure database [9] and Protein Data Bank in Europe—Knowledge Base (PDBe-KB) Database [10]. We employed the Discovery Studio Visualizer (v19.1) to refine further and construct the molecular structures.

### 2.2. Ligand Identification and Quantitative Structure–Activity Relationship (QSAR) Modeling

To identify the ligands that interact with AD-related protein targets, we extensively searched multiple databases, including ChEMBL [11], PubChem [12], ChemSpider [13], BindingDB [14], and PDBbind [15]. We identified the biological activity of each ligand based on the IC_50_ (nM) value, the concentration of the ligand required to inhibit a specific biological function by 50%. Next, we generated molecular fingerprints from the Simplified Molecular-Input Line-Entry System (SMILES) strings of each ligand using the RDKit cheminformatics toolkit [16]. The aggregated fingerprint data constituted the characteristic matrix utilized in our QSAR modeling approach. Following conventional protocols, the dataset underwent random partitioning into training and validation cohorts at an 80:20 ratio. The implementation of random forest regression techniques facilitated the optimization of predictive accuracy. To enhance model robustness and generalizability, *k*-fold cross-validation procedures were integrated during model training. From the IC50 of the NCs for AD-related targets, we calculated pIC_50_ values for training using the formula pIC_50_ = −log_10_ (IC_50_ × 10^−9^). We computed the residuals within the training set to evaluate model performance and identify outliers, representing the difference between the experimental and predicted values. We established a cutoff threshold at the 95th percentile of these residuals, enabling the identification and exclusion of compounds exceeding this threshold from further predictive analysis.

### 2.3. Prediction of Natural Compounds (NCs) Targeting AD-Related Proteins

We utilized OptNCMiner [7], a deep-learning method suitable for predicting optimal NCs with potential effects on the target proteins linked to AD (accessible at https://github.com/phytoai/OptNCMiner; 5 January 2024). Data on the food sources and the NCs derived from them were gathered from FooDB (https://foodb.ca; 18 November 2023). To form the training dataset, we categorized the interacting ligand dataset obtained from the aforementioned method into positive and negative pairs. Positive interactions were encoded as 1, while non-interactions were denoted as 0. Cosine similarity calculations between ligand pairs were implemented in the model architecture to facilitate the identification of NCs with potential protein-modulatory capabilities. Ligand pairs exhibiting cosine similarity metrics above 0.5 were designated as potentially bioactive. The FooDB repository was utilized to elucidate the dietary sources of the predicted NCs. The distribution patterns of NC origins were visualized through an interactive Venn diagram generated via the jvenn JavaScript library [17]. Furthermore, the biological efficacy of NCs targeting AD-associated proteins underwent systematic evaluation for stability and functional activity. The DeepChem (https://github.com/deepchem; 22 January 2024) framework was employed to transform chemical structural data into machine learning-compatible formats, utilizing pIC_50_ values from AD protein–ligand interactions.

### 2.4. Clustering and SAR Analysis of NCs

To assess the biological activities of the NCs associated with Alzheimer’s disease (AD), we employed clustering and SAR analysis. The Elbow and Silhouette Score methods were used to determine the optimal number of clusters, facilitating the grouping of NCs with similar biological activity profiles [18]. The Elbow method identifies the point where additional clusters provide diminishing improvements in within-cluster variance, while the Silhouette Score evaluates the consistency of clustering and separation between groups. Together, these methods ensured the robust and biologically meaningful categorization of NCs. Following clustering, we conducted an SAR analysis to explore the relationship between the structural and physicochemical properties of NCs and their biological activities. Molecular descriptors, which quantitatively represent structural features, were used to examine how specific structural modifications influence therapeutic efficacy in AD-related pathways. This approach highlighted key molecular features critical for modulating NC activity, providing insights into their mechanisms of action in targeting AD-associated pathways.

### 2.5. Comparative Analysis of Structural Properties and Molecular Commonality

A classification scheme was established for pIC_50_ values to facilitate structural analysis: low-potency (pIC_50_ ≤ 5), intermediate-potency (5 < pIC_50_ ≤ 7), and high-potency (pIC_50_ > 7) categories. This stratification enabled the systematic evaluation of structural variations relative to biological efficacy. The implementation of the RDKit maximum common substructure (MCS) algorithm revealed conserved molecular features among NCs exhibiting diverse activity profiles. The analysis of shared structural elements aimed to identify key molecular determinants of biological activity. The examination of preserved structural characteristics across potency levels revealed recurring molecular motifs associated with enhanced effectiveness against AD-related targets. Additionally, a functional group analysis across activity categories elucidated molecular components crucial for modulating NC activity against AD-specific targets.

### 2.6. Prediction of the Blood–Brain Barrier (BBB) Permeability

To efficiently screen potential central nervous system (CNS) drug candidates, a predictive method based on physicochemical properties and a scoring system is utilized to assess compounds’ ability to cross the BBB. This approach converts SMILES strings into molecular objects using the RDKit library, which are then analyzed to calculate physicochemical and BBB-specific descriptors. The BBB permeability score is derived from these properties, with compounds achieving a perfect score if they meet the following five criteria: (1) molecular weight ≤ 400 Da; (2) LogP value between −0.5 and 5; (3) hydrogen bond donors ≤ 3; (4) hydrogen bond acceptors ≤ 7; (5) topological polar surface area (TPSA) ≤ 90 Å^2^. This method provides a systematic and efficient means of identifying promising CNS drug candidates by focusing on critical molecular characteristics required for BBB penetration.

### 2.7. Molecular Docking and Binding Energy Analysis

The examination of potential NC-protein interactions in the context of AD commenced with the structural preparation of identified NCs and their corresponding protein targets. Molecular docking validation of the predicted interactions was executed using Autodock Vina [19]. This protocol encompassed the optimization of NC three-dimensional conformations and charge distribution calculations, alongside protein structure refinement. A semi-flexible docking approach was implemented, permitting ligand flexibility while maintaining protein receptor rigidity. Docking coordinates were established at the co-crystallized ligand binding site. Discovery Studio Visualizer (v19.1) and Chimera [20] facilitated the visualization of the docking outcomes. Hydrogen bond parameters were defined by specific geometric criteria: O-H distances below 2.50 Å, minimum angles of 120°, and centrally positioned E2 within the crystal structure. Interaction characterization incorporated binding energy calculations, accounting for molecular conformation, charge distribution, bond angles, and hydrogen bonding patterns. Molecular conformations underwent energy-based scoring and subsequent filtration to identify optimal protein-binding configurations. A comparative analysis of binding energies across bioactivity levels enabled the assessment of interaction strength between target proteins and NCs.

### 2.8. Validation Through In Vitro Study

The cells of the PC12 adherent cell line (ATCC CRL-1721.1) were cultured in Dulbecco’s Modified Eagle’s Medium (DMEM) (Sigma, Aldrich, St. Louis, MO, USA), supplemented with 10% horse serum, 5% fetal bovine serum, and 1% antibiotic mixture containing penicillin–streptomycin, in a humidified atmosphere at 37 °C with 5% CO_2_. The PC12 cells were seeded in 96-well plates at a density of 1 × 10^4^ cells/well and differentiated with 100 ng/mL nerve growth factor (NGF, Sigma-Aldrich, St. Louis, MO, USA) for 5 days into neuronal cells.

After the NGF-induced differentiation, the cells were exposed to 1 μg/mL lipopolysaccharide (LPS, Sigma-Aldrich) for 24 h to induce an inflammatory response. Subsequently, the cells were treated with different NCs (experimental groups) and vehicles (control), and the cells without LPS treatment were called the normal-control group. The NCs were various flavonoid compounds, including astragalin, dihydromyricetin, coumarin, quercetin, luteolin, chrysin, kaempferol, and apigenin (Sigma-Aldrich), at concentrations of 0, 1, 5, 25, and 25 μM. The flavonoid compounds were dissolved in dimethyl sulfoxide (DMSO, Sigma-Aldrich) and diluted in a culture medium, with the final DMSO concentration not exceeding 0.1%. After 24 h of flavonoid treatment, cell viability was assessed using a thiobarbituric acid reactive substances (TBARS) lipid peroxidation assay kit (DoGenBio, Seoul, Republic of Korea). Cell viability was expressed as a percentage relative to the untreated control cells.

NGF-induced differentiated cells exposed to LPS in 6-well plates (2 × 10^5^ cells/well) were treated with flavonoid compounds (0, 5, and 25 μM) for 48 h. Lipid peroxidation and acetylcholinesterase activity were measured using TBARS and acetylcholinesterase (AChE) assay kits (DoGenBio, Seoul, Republic of Korea), respectively. The levels of tumor necrosis factor-alpha (TNF-α) and interleukin (IL)-1β were determined using enzyme-linked immunosorbent assay (ELISA kits, R & D Systems, Minneapolis, MN, USA, and Abcam, Cambridge, MA, USA, respectively). 

### 2.9. Analysis of Gene Expression

The total RNA of the cells was extracted using TRIzol reagent (Invitrogen, Carlsbad, CA, USA) and reverse transcribed to cDNA using a high-capacity cDNA reverse transcription kit (Applied Biosystems, Foster City, CA, USA). Quantitative real-time PCR was performed using the SYBR Green master mix (Bio-Rad, Hercules, CA, USA) on a CFX96 real-time PCR system (Bio-Rad). The gene expression levels of *TNF-α*, brain-derived neurotrophic factor (*BDNF*), and ciliary neurotrophic factor (*CNTF*) were normalized to glyceraldehyde 3-phosphate dehydrogenase (*GAPDH*) and calculated using the 2^−ΔΔCt^ method.

### 2.10. Statistical Analysis

Data were presented as the mean ± standard deviation (SD) from three independent experiments. One-way ANOVA with Tukey’s post hoc test was used for multiple comparisons. *p* < 0.05 was considered statistically significant.

## 3. Results

### 3.1. QSAR Modeling for the Evaluation of Prediction Accuracy

**Figure 1:**
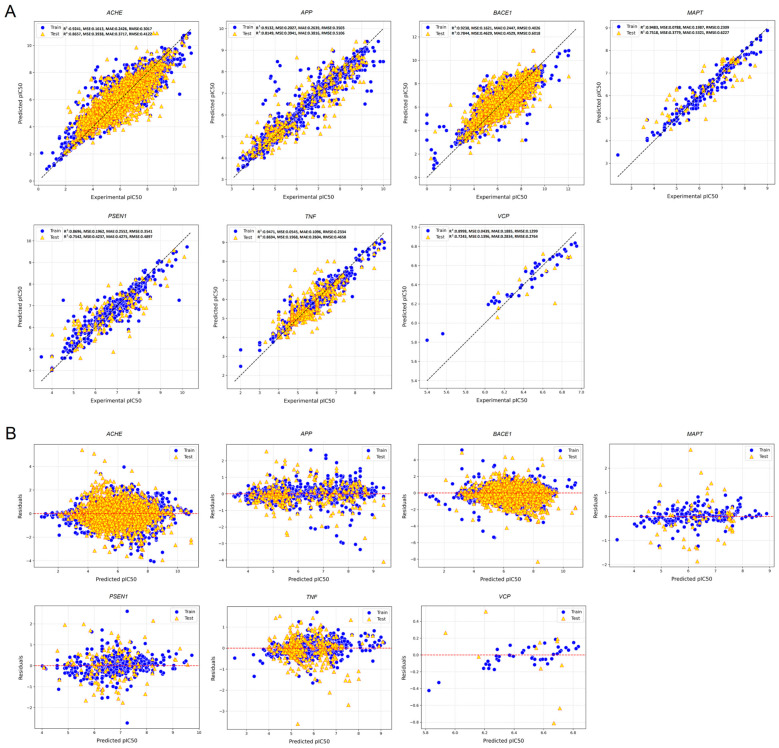
Assessment of predictive accuracy and residual analysis for the pIC_50_ values of ligands interacting with AChE, APP, BACE1, MAPT, PSEN1, TNF-α, and VCP using a random forest regression model. (**A**) Scatter plots juxtapose the predicted and experimental pIC_50_ values for ligands interacting with AChE, APP, BACE1, MAPT, PSEN1, TNF-α, and VCP in both the training (depicted as blue circles) and testing (illustrated as yellow triangles with red edges) datasets. Performance metrics, including mean squared error (MSE), R^2^ value, mean absolute error (MAE), and root mean squared error (RMSE), offer insights into the model’s prediction accuracy. (**B**) The residual analysis highlights disparities between the model-predicted and experimental pIC_50_ values. The blue circles denote discrepancies in the training dataset, and the yellow triangles with red edges signify variations in the testing dataset. The red dashed line represents the theoretical ideal of zero residuals, indicating a perfect alignment between the model’s predictions and experimental results. AChE, acetylcholinesterase; APP, amyloid precursor protein; BACE1, beta-secretase 1; MAPT, microtubule-associated protein tau; PSEN1, presenilin-1; TNF-α, tumor necrosis factor; VCP, valosin-containing protein.

**Figure 2:**
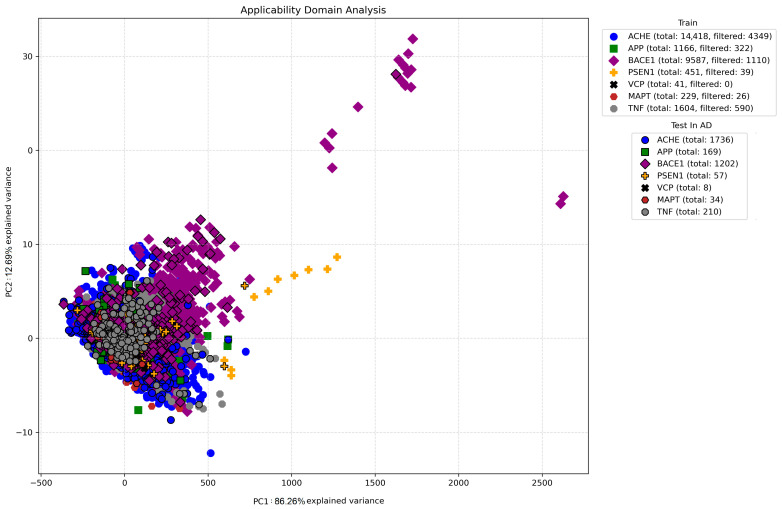
Applicability domain analysis of the interacting ligands via the principal component analysis (PCA). This figure utilizes PCA to assess the dataset variance of compounds interacting with AChE, APP, BACE1, MAPT, PSEN1, TNF-α, and VCP. Each point signifies an interacting ligand positioned by molecular fingerprints, with distinct markers and colors indicating the spread and concentration in the training dataset. The 95% quantile of residuals serves as a cut-off, identifying outliers. The test dataset emphasizes compounds within the applicability domain with a unique edge color. The x and y axes represent the first and second principal components, annotated with variance proportions. PCA, principal component analysis; AChE, acetylcholinesterase; APP, amyloid precursor protein; BACE1, beta-secretase 1; MAPT, microtubule-associated protein tau; PSEN1, presenilin-1; TNF-α, tumor necrosis factor; VCP, valosin-containing protein.

We selected seven specific AD-related genes, including acetylcholinesterase (*AChE*), amyloid precursor protein (*APP*), beta-secretase 1 (*BACE1*), microtubule-associated protein tau (*MAPT*), presenilin-1 (*PSEN1*), tumor necrosis factor (*TNF*)*-*α, and valosin-containing protein (*VCP*), which had high relevance scores. The relevance scores of the selected genes are presented in Supplementary Table S1. The proteins encoded by these genes were known to interact with ligands in the pathological cascade of AD. These interacting ligands were identified for each target protein. The number of interacting ligands for the target proteins was as follows: 16,154 for *AChE*, 1335 for *APP*, 10,789 for *BACE1*, 508 for *MAPT*, 49 for *PSEN1*, 263 for *TNF-α*, and 1814 for *VCP*. In the random forest regression models to predict the pIC_50_ values for the interacting ligands with the selected targets, the mean square error (MSE) results for each target were computed, yielding the values of 0.1613 for *AChE*, 0.2027 for *APP*, 0.1621 for *BACE1*, 0.0788 for *MAPT*, 0.1962 for *PSEN1*, 0.0545 for *TNF-α*, and 0.0439 for *VCP*, respectively (Figure 1A). These low MSE values indicate a minimal disparity between the predicted and observed pIC_50_ values, underscoring the precision of the predictive models (Figure 1A). Remarkably, the results revealed no discernible pattern between the experimental and predicted pIC_50_ values in residual analysis, with residuals primarily clustering around the zero line. This observation also indicates the absence of systematic errors, underscores the robustness and reliability of the predictive models, and suggests their high accuracy (Figure 1B). The principal component analysis (PCA) to find a suitability domain encompassed a comprehensive dataset comprising 30,912 interacting ligands, selected based on a stringent pIC_50_ threshold (5.0). Subsequently, a cutoff value for filtering out potential outliers was set at the 95th percentile of residuals derived from the predictive models. This process excluded 6436 ligands, thereby refining the dataset to the final 24,476 interacting ligands deemed suitable for participating in subsequent predictions by deep neural analysis (Figure 2).

### 3.2. Predictions by Deep Neural Analysis and Food Source Analysis

**Figure 3:**
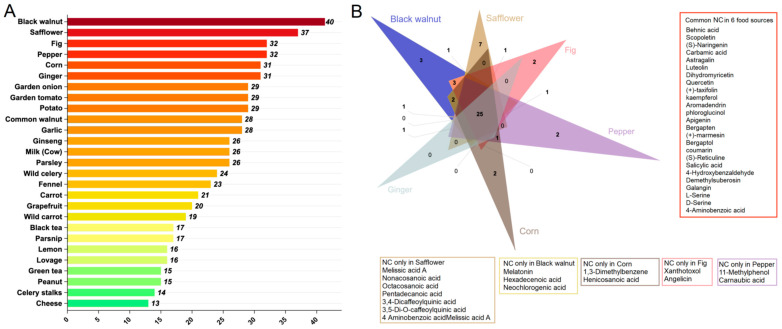
Distribution and overlap of natural compounds (NCs) in various food sources. (**A**) A bar chart provides specific information on food sources with more than 10 NCs. (**B**) An interactive Venn diagram illustrates the distribution and overlap of NCs across the selected food sources, offering insights into their presence and shared compounds. Each Venn diagram section corresponds to a specific food source, with overlapping regions highlighting shared NCs.

NCs were predicted to have potential effects on AD in seven proteins, as detailed in Supplementary Table S2. The predictions generated by the deep learning models demonstrated outstanding recall performance, underscoring their efficacy in identifying the potential NCs associated with AD. The top six food sources of these predicted NCs were selected. Notably, black walnut emerged as a prominent source, yielding 40 NCs, followed closely by safflower with 37 NCs. Additionally, fig and pepper served as sources of 32 NCs each, while corn and ginger contributed 31 NCs (Figure 3A). Further analysis unveiled 25 common NCs across the top six food sources (Figure 3B). These compounds included behenic acid, scopoletin, (S)-naringenin, carbamic acid, astragalin, luteolin, dihydromyricetin, quercetin, (+)-taxifolin, kaempferol, aromadendrin, phloroglucinol, apigenin, bergapten, (+)-marmesin, bergaptol, coumarin, (S)-reticuline, salicylic acid, 4-hydroxybenzaldehyde, demethylsuberosin, galangin, L-serine, D-serine, and 4-aminobenzoic acid (Figure 3B).

### 3.3. Clustering Analysis of NCs

**Figure 4:**
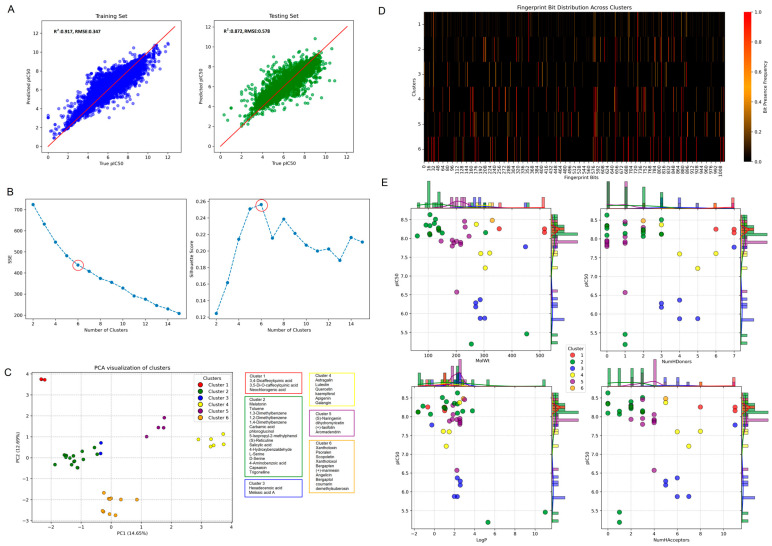
Model performance, optimal clustering, and structure–activity relationships in the prediction of bioactivity of natural compounds (NCs). (**A**) Scatter plots assess the model’s predictive performance on training (**left**) and testing (**right**) datasets for the pIC_50_ values. R^2^ and root mean squared error (RMSE) metrics quantify predictive accuracy. (**B**) Optimal cluster count determination using the Elbow and Silhouette Score methods. The Elbow method identifies the best number of clusters based on the rate of decrease in the sum of squared errors (SSE), while the Silhouette Score method gauges cluster quality. Red circle indicated the optimal number of the clusters. (**C**) Scatter plot visualizes the K-Means clustering of compounds based on molecular attributes and biological activity. (**D**) The heatmap displays fingerprint bit distribution across the K-Means clusters, revealing patterns and similarities. (**E**) Structure–activity relationship (SAR) plots illustrate various molecular descriptors against IC_50_ values, color-coded by cluster assignment.

Using the pIC_50_ values of 24,476 interacting ligands, we employed the DeepChem model to predict the biological potency of 41 NCs sourced from the top five food sources. The scatter plot depicted in Figure 4A illustrates the performance of the DeepChem model on both the training (left) and test (right) datasets. Notably, the model exhibited high prediction accuracy, achieving an R^2^ value of 0.917 and a root mean square error (RMSE) value of 0.347 for the training dataset and an R^2^ value of 0.872 and an RMSE value of 0.578 for the test dataset. Following the bioactivity prediction, we clustered the NCs to discern their distinct structural and chemical characteristics. Based on the determination of six as the optimal number of clusters (Figure 4B), a scatter plot (Figure 4C) was employed to illustrate the distribution and activity levels of the NCs within each cluster. This visualization identified six distinct clusters characterized by comparable structural and chemical attributes. Subsequently, an SAR analysis was conducted, focusing on various molecular descriptors associated with the pIC_50_ values, which serve as an indicator of compound potency. For a comprehensive understanding of the SAR, we compiled detailed data in Supplementary File S3, including the SMILES strings, pIC50 values, and target names of the natural compounds involved in our study. The SAR analysis results, presented in Figure 4D, demonstrate differences in molecular weight (MolWt), hydrogen donor count (NumH Donors), and hydrogen acceptor count (NumH Acceptors) across the clusters. These results emphasize the strong correlation between the molecular characteristics and the biological activity of the NCs within each cluster.

### 3.4. Classification and Common Functional Group Analysis of NCs

**Figure 5:**
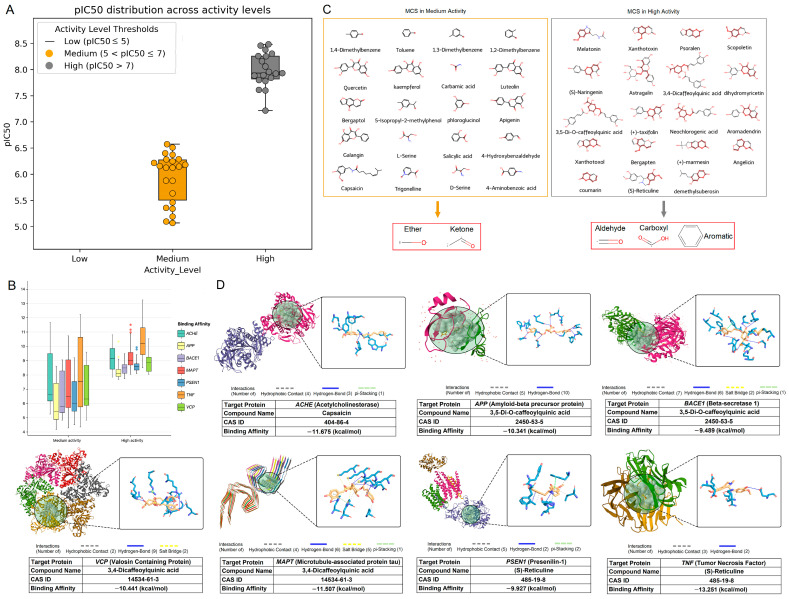
Bioactivities and interactions of natural compounds (NCs) at different levels. (**A**) pIC_50_ distribution across activity levels. This visualization shows pIC_50_ distribution across ‘low’, ‘medium’, and ‘high’ activity levels. Box plots depict interquartile range (IQR) with median lines, and whiskers extend to data points within 1.5 times the IQR. Swarm plots represent individual NCs with high affinity, color-coded for medium (5 < pIC_50_ ≤ 7) and high activity (pIC_50_ > 7). (**B**) Molecular docking: The NCs’ molecular docking results with target proteins, emphasizing stronger affinity with lower negative energy. (**C**) Maximum common substructures (MCSs) across activity levels: Panels segregate NCs based on bioactivity levels, showing molecular structures with highlighted maximum common substructures (MCSs). Each grid represents a specific activity level, providing insights into the common functional groups affecting bioactivity. (**D**) Detailed visualization of the highest affinity interaction: provides a detailed view of the interaction between the target protein and the highest affinity natural compound, offering a complex visualization of specific interactions.

NCs were classified according to their activity levels, where higher pIC_50_ values corresponded to greater biological activity. As shown in Figure 5A, the analysis indicated the absence of NCs with low activity levels. Specifically, 22 NCs were categorized as moderately active (5 < pIC_50_ ≤ 7), while 19 NCs were identified as highly active (pIC_50_ > 7). The binding energy in the molecular docking between the target protein and the selected NCs demonstrated that the NCs with higher activity exhibited a greater binding affinity among the seven target proteins than those with moderate activity (Figure 5B). Detailed docking information is provided in Supplementary Table S3. Upon the analysis of the NCs with moderate activity, we observed common functional groups within their structures, specifically the ether and ketone groups. Conversely, NCs with high activity levels predominantly featured the aldehyde, carboxyl, and aromatic functional groups. Further examination revealed the NCs exhibiting the lowest binding energy among the seven target proteins, indicating the highest binding affinities between them. Notably, capsaicin exhibited the highest affinity when docked with AChE at −11.675 kcal/mol, while 3,5-di-O-caffeoylquinic acid displayed strong affinity with APP at −10.342 kcal/mol. Additionally, 3,4-dicaffeoylquinic acid and (S)-reticuline exhibited notable affinities with BACE1, MAPT, PSEN1, TNF-α, and VCP, respectively. Supplementary Figure S1 presents the NCs to satisfy these criteria for BBB permeability. Among the six NCs for in vitro studies, BBB permeability percentages ranged from 40% to 100%, as follows: astragalin (60%), dihydromyricetin (40%), coumarin (100%), quercetin (60%), kaempferol (60%), apigenin (100%), and luteolin (60%). These results suggest that the six selected NCs hold significant potential for further investigation in AD functional food development.

### 3.5. In Vitro Study

**Figure 6:**
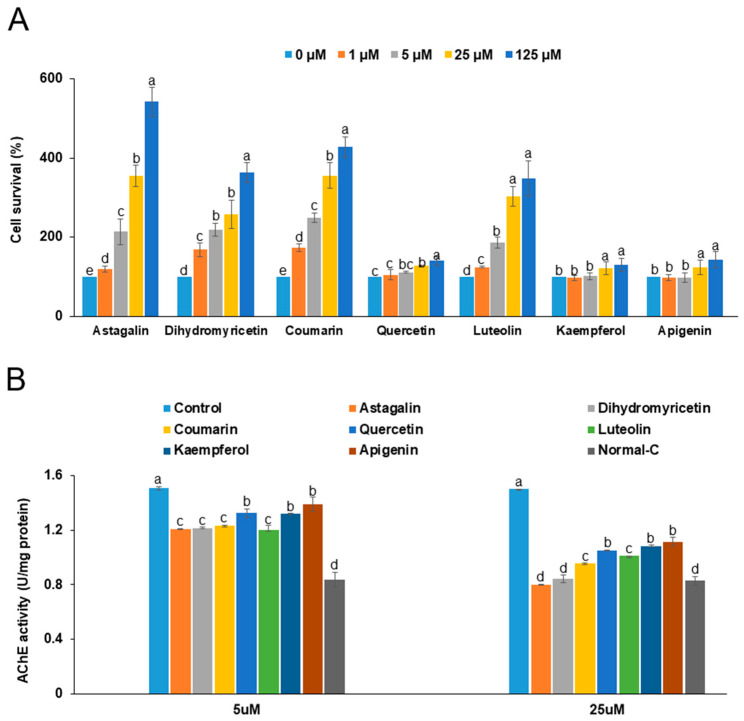
Cell viability (**A**) and acetylcholinesterase (AChE) activity (**B**) Nerve growth factor (NGF)-differentiated PC12 cells with lipopolysaccharide-induced inflammation (LPS, 1 μg/mL) were treated with astragalin, dihydromyricetin, coumarin, quercetin, kaempferol, apigenin, and luteolin for 24 h to measure cell viability and 48 h to measure AChE activity. a–e Different letters on the bar indicated significant differences between the groups at *p* < 0.05.

Cell viability in the NGF-differentiated PC12 cells was significantly reduced following the administration of 1 µg/mL LPS compared to the cells without LPS treatment. However, treatment with astragalin, dihydromyricetin, coumarin, quercetin, kaempferol, apigenin, and luteolin at concentrations of 0, 1, 5, 25, and 125 µM led to an increase in cell viability (Figure 6A). Notably, astragalin, dihydromyricetin, and coumarin, which were classified in the high-activity group, promoted cell viability in a dose-dependent manner (Figure 6A). In the medium-activity group, quercetin, kaempferol, apigenin, and luteolin also enhanced cell viability, although to a lesser extent than the high-activity compounds. Interestingly, luteolin demonstrated a cell viability enhancement comparable to that of the high-activity group.

The LPS treatment led to an increase in acetylcholinesterase (AChE) activity, which is associated with memory deficits due to reduced acetylcholine levels (Figure 6B). Treatment with astragalin, dihydromyricetin, coumarin, quercetin, kaempferol, apigenin, and luteolin (at concentrations of 5 and 25 µM) resulted in a decrease in AChE activity compared to the control group. Specifically, the 25 µM concentrations of astragalin, dihydromyricetin, coumarin, and luteolin significantly reduced AChE activity more than the normal-control group, whereas the decrease in AChE activity induced by quercetin, kaempferol, and apigenin was similar to that observed in the normal-control group.

**Figure 7:**
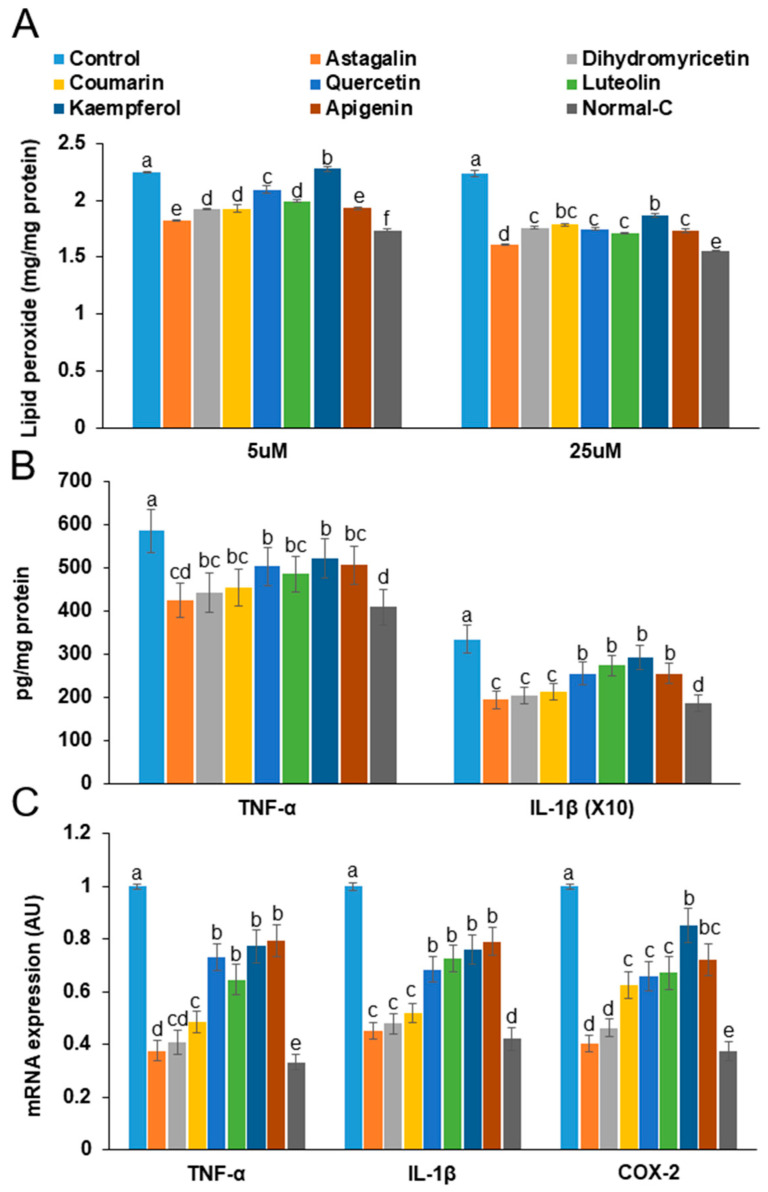
Lipid peroxidation (**A**) and protein (**B**) and mRNA expression (**C**) of pro-inflammatory cytokines. Nerve growth factor (NGF)-differentiated PC12 cells with lipopolysaccharide-induced inflammation (LPS, 1 μg/mL) were treated with astragalin, dihydromyricetin, coumarin, quercetin, kaempferol, apigenin, and luteolin for 48 h. a–e Different letters on the bar indicated significant differences between the groups at *p* < 0.05.

To assess lipid peroxidation, malondialdehyde (MDA) levels were measured in NGF-differentiated PC12 cells. Treatment with astragalin, dihydromyricetin, coumarin, quercetin, kaempferol, apigenin, and luteolin resulted in a dose-dependent reduction in lipid peroxidation compared to the control group. However, the levels of lipid peroxidation were still higher than those in the normal-control group. Among the compounds tested, astragalin was the most effective in reducing lipid peroxidation (Figure 7A). Furthermore, the mRNA expression levels of pro-inflammatory cytokines TNF-α and IL-1βwere significantly decreased following treatment with astragalin, dihydromyricetin, coumarin, quercetin, kaempferol, apigenin, and luteolin compared to the control. Dihydromyricetin reduced these levels to a similar extent as the normal-control group (Figure 7B). The mRNA expression of TNF-α and IL-1β displayed a similar trend to that observed for the protein expression, with the effect on mRNA levels being more pronounced than the changes in protein levels (Figure 7C).

## 4. Discussion

Our bioinformatics-integrated deep neural analysis results provide promising insights into the therapeutic potential of NCs in AD. Identifying compounds that interact with key AD-related targets, such *as AChE*, *APP*, *BACE1*, *MAPT*, *PSEN1*, *TNF-α*, and *VCP*, highlights their probable influence on critical pathways central to AD pathogenesis. These include the pathways related to amyloid-beta production, neuroinflammation, and neuronal damage, which are hallmarks of AD. Molecular docking analysis demonstrated that the selected NCs exhibited reduced binding energies with their target proteins, suggesting potential efficacy in modulating AD-related pathological processes. Furthermore, preliminary in vitro experiments revealed that these compounds exert beneficial effects in LPS-induced inflamed neuronal cells, as evidenced by enhanced cell survival, reduced lipid peroxidation, and suppressed pro-inflammatory cytokine production. These findings underscore the multi-target potential of NCs in mitigating the complex pathological processes associated with AD. The development of therapeutic strategies capable of simultaneously addressing multiple targets involved in AD pathogenesis holds significant promise. While further validation through animal studies and human trials is necessary to confirm the efficacy and safety of these compounds, our findings lay a solid foundation for future research. The integration of computational and experimental methods in this study highlights a novel and efficient approach to accelerating the identification of NCs with disease-modifying potential for AD, paving the way for more effective therapeutic interventions.

The present study introduces a novel bioinformatics-integrated deep neural analysis approach, OptNCMiner, representing a significant methodological advancement over traditional drug discovery techniques. Unlike conventional screening methods or structure-based approaches widely employed in earlier research [21], OptNCMiner allows for the exploration of the therapeutic potential of NCs across multiple gene targets simultaneously. This methodology has already shown promise in previous studies on metabolic diseases, including AD, where it was used to investigate the therapeutic effects of NCs on multi-target genes [7,22]. By leveraging this approach, the current study is able to overcome the limitations of traditional methods, providing a more comprehensive and effective strategy for drug discovery. Our methodology leverages the integration of large-scale chemical and biological data from databases like ChEMBL and functional food databases in combination with AD-related gene information based on relevance scores. Moreover, a key strength of our approach, compared to previous studies, is the utilization of advanced computational techniques, such as deep learning models (random forest regression), for accurately predicting the potential activity (pIC_50_ values) of compounds against AD-related targets [23,24,25]. This data-driven approach expands chemical space exploration, enabling the identification of novel therapeutic candidates that may have been overlooked by the traditional methods employed in earlier studies [21,26].

Our methodology considered multiple key AD-related genes (*AChE*, *APP*, *BACE1*, *MAPT*, *PSEN1*, *TNF-α*, and *VCP*) and their proteins, which is a significant advantage over previous studies that focused on a limited set of targets or employed simplistic scoring functions [26,27]. By considering compounds with potential activity across multiple targets [28], our study increases the likelihood of identifying compounds with diverse mechanisms of action, which could lead to more effective therapeutic interventions suitable for the complex and multifaceted nature of AD. Unlike previous studies that relied solely on computational predictions or in vitro experiments [29,30], the present study incorporates both molecular docking analysis and in vitro neuronal cell experiments, providing a more comprehensive validation of the identified compounds. This integrated approach allows for the assessment of binding interactions between the compounds and their targets, as well as insights into their neuroprotective and disease-modifying potential in a biologically relevant context.

Among the NCs identified through the bioinformatics-integrated deep neural analysis in the present study, the effects of specific compounds were confirmed in vitro. Astragalin, dihydromyricetin, and coumarin in the high-activity group and luteolin in the medium-activity group identified through the prediction model improved cell viability in LPS-induced inflamed neuronal cells. Previous studies have also reported their protective effects on AD. Astragalin, a flavonoid, has demonstrated neuroprotective and anti-inflammatory properties. It has been shown to inhibit Aβ aggregation, reduce oxidative stress, protect neuronal cells from Aβ-induced toxicity in vitro, improve cognitive function, and reduce Aβ deposition in transgenic AD mice models [31,32]. Similarly, dihydromyricetin has been shown to inhibit Aβ aggregation, reduce oxidative stress, protect neuronal cells from Aβ toxicity in vitro, improve cognitive function, reduce Aβ deposition, and alleviate neuroinflammation in AD mouse models [33,34]. Coumarin, a plant-derived compound, inhibits Aβ aggregation, reduces oxidative stress, protects neuronal cells from Aβ toxicity in vitro, improves cognitive function, and reduces Aβ deposition in AD mouse models [35]. Luteolin, a flavonoid, has been extensively studied for its potential therapeutic effects in AD, demonstrating the inhibition of Aβ aggregation, reduction in oxidative stress, protection of neuronal cells from Aβ toxicity in vitro, as well as the improvement of cognitive function, decline in Aβ deposition, and alleviation of neuroinflammation in AD mouse models [4,36]. These findings validate the appropriateness of exploring AD therapeutic agents through bioinformatic-based analysis, considering their interactions with multiple target proteins.

The principal advantage of this research is attributed to the implementation of an innovative bioinformatics-based deep neural analysis approach for the screening of numerous NCs against a spectrum of AD-related targets. This approach has facilitated the discovery of potential novel therapeutic agents that may have been disregarded by traditional screening methods, owing to the utilization of extensive data amalgamation and sophisticated computational algorithms. Furthermore, targeting multiple pathways is deemed more holistic, considering the intricate and multi-dimensional characteristics of AD etiology, thereby enhancing the probability of uncovering NCs with a range of therapeutic mechanisms. Another strength is the comprehensive validation process, which combined molecular docking analysis with functional in vitro experiments using neuronal cell models. This integrated approach provided insights into both the binding interactions between compounds and their targets, as well as their functional effects in a biologically relevant context, strengthening the credibility of the identified natural compounds. Our methodology is not limited to AD; it is also highly adaptable for studying other complex diseases with multifaceted molecular mechanisms. We now provide guidelines for implementing this approach in other research contexts, highlighting its interdisciplinary potential and the ability to integrate into various fields of study. This approach offers new perspectives and valuable tools for investigating a broad range of diseases. Furthermore, the scalability of our methodology allows for its application to larger datasets and more complex disease models, increasing its broader applicability. It is important to note that while several promising NCs identified in this study, such as astragalin, dihydromyricetin, coumarin, and luteolin, have been previously explored for their therapeutic effects in AD, our approach effectively demonstrates the ability to systematically evaluate their multi-target interactions and prioritize them for further research.

This study has several limitations. While the deep neural network-based prediction models demonstrated excellent recall performance, their accuracy may have been influenced by the quality and completeness of the training data. Additionally, PC12 cells differentiated with NGF, which possess sympathetic nerve-like characteristics and catecholamine-releasing capabilities, offer a useful model for studying neuronal morphology and functions [37]. However, these in vitro experiments may not fully replicate the complex physiological environment of human AD patients, particularly the challenges posed by the BBB. The ability of NCs to cross the BBB is a critical factor for their therapeutic potential in treating CNS disorders. In this study, the predicted BBB permeability percentages of the six selected NCs ranged from 40% to 100%, suggesting potential as functional candidates for AD. However, more comprehensive investigations are needed, including evaluating the mechanisms of transport across the BBB and exploring delivery strategies to enhance their accessibility to CNS targets. Furthermore, future studies should assess whether these compounds can be metabolized by gut microbiota to form BBB-permeable derivatives or exert indirect effects on the brain via the gut–brain axis. Notably, compounds such as luteolin and dihydromyricetin, which demonstrated relatively lower predicted BBB penetration (60% and 40%, respectively), have shown memory-enhancing effects in previous animal studies [4,34,36], underscoring the importance of investigating alternative mechanisms of action. Despite these limitations, our study establishes a solid foundation for bioinformatics-integrated approaches to accelerate the discovery of novel therapeutic agents for AD. It highlights the potential of NCs as promising candidates for future development, with the need for further validation in animal models and clinical settings.

## 5. Conclusions

This study demonstrated the power of a bioinformatics-integrated deep neural analysis approach in identifying promising NCs for AD therapy. By leveraging large-scale data integration, advanced computational techniques, and multi-target considerations, our methodology addressed some of the limitations of traditional drug discovery methods. The systematic evaluation of NCs against multiple AD-related targets corroborated the therapeutic potential of previously studied compounds like astragalin, dihydromyricetin, coumarin, and luteolin. It highlighted other promising candidates worthy of further investigation. As understanding disease mechanisms and the availability of large-scale data continues to evolve, such integrative approaches will become increasingly valuable in accelerating the drug discovery process and ultimately improving patient outcomes.

## Data Availability

The original contributions presented in the study are included in the article/Supplementary Material, further inquiries can be directed to the corresponding author.

## Supplementary Materials

The following supporting information can be downloaded at https://www.mdpi.com/article/10.3390/foods14010127/s1, Table S1. The relevance scores of the selected genes for Alzheimer’s disease. Table S2. Potential activities of 166 natural compounds on proteins related to Alzheimer’s disease. Table S3. Docking information between Alzheimer’s disease-related protein and natural compounds. Supplementary Figure S1. Percentage of BBB permeability in natural compounds. Supplementary File S1. CAS numbers, smiles, and food sources of the selected natural compounds. File S2. Alzheimer’s disease-related proteins, and smiles of natural compounds, and their docking scores. File S3. Structure–activity relationship (SAR) analysis data—SMILES, pIC_50_ values, and target information of natural compounds.

## Abbreviations

AD, Alzheimer’s disease; BioDeepNat, bioinformatics-integrated deep neural analysis of natural compounds for Disease Discovery; *AChE*, acetylcholinesterase; *APP*, amyloid-β precursor protein; *BACE1*, beta-secretase 1; *MAPT*, microtubule-associated protein tau; *PSEN1*, presenilin-1; *TNF*, tumor necrosis factor; *VCP*, valosin-containing protein; LPS, lipopolysaccharide; *PSEN*, presenilin; *APOE*, apolipoprotein E; *TREM2*, triggering receptor expressed on myeloid cells 2; *SIGLEC-3*, sialic acid-binding immunoglobulin-type of lectins; *SORL1*, sortilin-related receptor 1; Aβ, amyloid-β peptide; EGCG, epigallocatechin-3-gallate; SMILES, Simplified Molecular-Input Line-Entry System; QSAR, quantitative structure–activity relationship; NCs, Natural Compounds; SAR, structure–activity relationship; MCS, maximum common substructure; DMEM, Dulbecco’s Modified Eagle’s Medium; NGF, nerve growth factor; TBARS, thiobarbituric acid reactive substances; IL, interleukin; BDNF, brain-derived neurotrophic factor; CNTF, ciliary neurotrophic factor; GAPDH, glyceraldehyde 3-phosphate dehydrogenase; SD, standard deviation; PCA, principal component analysis; RMSE, root mean square error; MDA, malondialdehyde; MSE, mean squared error
