# Supplementary material for: Bioinformatics and Deep Learning Approach to Discover Food-Derived Active Ingredients for Alzheimer’s Disease Therapy
Source: Foods. 2025 Jan 4;14(1):127. doi: 10.3390/foods14010127 (PMC11719994; doi:10.3390/foods14010127)
Supplement: Supplementary file 1 [file foods-14-00127-s001.zip › supplementary tables.pdf]

Supplementary Table S1. The relevance scores of the selected genes for Alzheimer's disease

	Symbol	Descriptio	Category	UniProt ID	GIFtS	GC id	Score ▼
1	VCP	Valosin Co	Protein Co	P55072	53	GC09M035	108.9
2	MAPT	Microtubu	Protein Co	P10636	53	GC17P045	97.98
3	GRN	Granulin P	Protein Co	P28799	51	GC17P044	87.17
4	SQSTM1	Sequestos	Protein Co	Q13501	53	GC05P179	80.82
5	PSEN1	Presenilin	Protein Co	P49768	55	GC14P073	79.1
6	FUS	FUS RNA E	Protein Co	P35637	48	GC16P031	76.17
7	TARDBP	TAR DNA	Protein Co	Q13148	51	GC01P011	71.96
8	C9orf72	C9orf72-SI	Protein Co	Q96LT7	44	GC09M029	70.39
9	GBA1	Glucosylce	Protein Co	P04062	53	GC01M158	62.84
10	SNCA	Synuclein	Protein Co	P37840	54	GC04M089	61.13
11	DNMT1	DNA Meth	Protein Co	P26358	56	GC19M010	60.01
12	TBK1	TANK Binc	Protein Co	Q9UHD2	52	GC12P064	56.86
13	APOE	Apolipopr	Protein Co	P02649	53	GC19P096	55.38
14	CHMP2B	Charged M	Protein Co	Q9UQN3	47	GC03P087	54.86
15	LOC10662	GBA Recor	Functional	Element	4	GC01P155	54.63
16	CHCHD10	Coiled-Coi	Protein Co	Q8WYQ3	42	GC22M023	54.48
17	HNRNPA2	Heterogen	Protein Co	P22626	50	GC07M026	52.39
18	UBQLN2	Ubiquilin 2	Protein Co	Q9UHD9	46	GC0XP056	48.2
19	TREM2	Triggering	Protein Co	Q9NZC2	48	GC06M087	46.98
20	APP	Amyloid B	Protein Co	P05067	54	GC21M025	45.95
21	CCNF	Cyclin F	Protein Co	P41002	44	GC16P002	39.89
22	PRNP	Prion Prot	Protein Co	F7VJQ1	52	GC20P004	39.29
23	SNCB	Synuclein	Protein Co	Q16143	47	GC05M176	38.88
24	OPTN	Optineurin	Protein Co	Q96CV9	47	GC10P013	36.75
25	NOTCH3	Notch Rec	Protein Co	Q9UM47	54	GC19M015	36.64
26	CSF1R	Colony Sti	Protein Co	P07333	55	GC05M150	36.05
27	SPTLC1	Serine Palr	Protein Co	O15269	50	GC09M105	35.5
28	ITM2B	Integral M	Protein Co	Q9Y287	48	GC13P048	34.17
29	TNF	Tumor Ne	Protein Co	P01375	55	GC06P118	33.68
30	ABCA7	ATP Bindir	Protein Co	Q8IZY2	47	GC19P094	33.03
31	HNRNPA1	Heterogen	Protein Co	P09651	51	GC12P054	32.73
32	TMEM106	Transmem	Protein Co	Q9NUM4	43	GC07P012	32.61
33	PARK7	Parkinson	Protein Co	Q99497	51	GC01P009	32.01
34	LOC12517	Sharpr-MP	Functional	Element	3	GC17P095	31.46
35	LOC10950	Chromoso	Functional	Element	3	GC09P027	30.67
36	DCTN1	Dynactin S	Protein Co	Q14203	50	GC02M074	30.34
37	SOD1	Superoxide	Protein Co	P00441	56	GC21P031	29.86
38	TRPM7	Transient F	Protein Co	Q96QT4	48	GC15M050	28.85
39	PSEN2	Presenilin	Protein Co	P49810	52	GC01P226	28.09
40	TIA1	TIA1 Cytot	Protein Co	P31483	46	GC02M070	27.58
41	SETX	Senataxin	Protein Co	Q7Z333	46	GC09M132	27.01
42	SLC25A24	Solute Car	Protein Co	Q6NUK1	46	GC01M108	26.89

43	MIR659	MicroRNA	RNA Gene		19	GC22M07	26.22
44	SERPINI1	Serpin Fan	Protein Co	Q99574	50	GC03P167	25.81
45	ERBB4	Erb-B2 Re	Protein Co	Q15303	58	GC02M211	25.62
46	ANG	Angiogeni	Protein Co	P03950	48	GC14P041	24.73
47	GLT8D1	Glycosyltra	Protein Co	Q68CQ7	41	GC03M05	24.7
48	BACE1	Beta-Secre	Protein Co	P56817	51	GC11M11	24.69
49	TUBA4A	Tubulin Al	Protein Co	P68366	51	GC02M21	24.67
50	ATXN2	Ataxin 2	Protein Co	Q99700	47	GC12M111	24.57
51	TYROBP	Transmem	Protein Co	O43914	46	GC19M03	23.94
52	CYLD	CYLD Lysi	Protein Co	Q9NQC7	51	GC16P050	23.28
53	MT-ND1	Mitochonc	Protein Co	P03886	34	GCMTP00	23.28
54	PRKN	Parkin RB	Protein Co	O60260	52	GC06M161	23.26
55	LRRK2	Leucine Ri	Protein Co	Q5S007	53	GC12P040	22.95
56	ATP13A2	ATPase Ca	Protein Co	Q9NQ11	48	GC01M01	22.93
57	CHRNA4	Cholinergi	Protein Co	P43681	53	GC20M06	21.56
58	SORL1	Sortilin Rel	Protein Co	Q92673	49	GC11P121	20.51
59	NEAT1	Nuclear P	RNA Gene		26	GC11P081	19.9
60	BDNF-AS	BDNF Anti	RNA Gene		22	GC11P027	19.67
61	TREX1	Three Prim	Protein Co	Q9NSU2	46	GC03P054	19.59
62	HTT	Huntingtin	Protein Co	P42858	48	GC04P003	19.26
63	MATR3	Matrin 3	Protein Co	P43243	46	GC05P139	19.05
64	LOC10890	10p13 OP	Functional Element		4	GC10P013	19.05
65	MIR22	MicroRNA	RNA Gene		23	GC17M001	19.02
66	BCHE	Butyrylcho	Protein Co	P06276	53	GC03M16	18.98
67	LOC12686	CDK7 Stro	Functional Element		3	GC19P097	18.65
68	PON1	Paraoxona	Protein Co	P27169	51	GC07M09	18.63
69	RNASE4	Ribonucle	Protein Co	P34096	38	GC14P041	18.36
70	EGILA	EGFR Inter	RNA Gene		15	GC14M02	18.36
71	ANXA11	Annexin A	Protein Co	P50995	47	GC10M08	18.28
72	RPS27A	Ribosomal	Protein Co	P62979	46	GC02P055	18.25
73	HTRA1	HtrA Serin	Protein Co	Q92743	48	GC10P122	18.19
74	MEF2C	Myocyte E	Protein Co	Q06413	51	GC05M08	18.19
75	ACHE	Acetylcholi	Protein Co	P22303	50	GC07M10	18.05

Table S2. Potential activities of 166 natural compounds on proteins related to Alzheimer's disease

ID_Uniprot	Protein	Descriptio	SMILES	Chemical Name
P22303	ACHE	Acetylcholi	CC(C)(O)C	(+)-marmesin
P22303	ACHE	Acetylcholi	O[C@@H	(+)-taxifolin
P22303	ACHE	Acetylcholi	[H][C@]1(	(S)-Naringenin
P22303	ACHE	Acetylcholi	COC1=C(	(S)-Reticuline
P22303	ACHE	Acetylcholi	CC1=CC=	1,2-Dimethylbenzene
P22303	ACHE	Acetylcholi	CC1=CC(	1,3-Dimethylbenzene
P22303	ACHE	Acetylcholi	CC1=CC=	1,4-Dimethylbenzene
P22303	ACHE	Acetylcholi	O[C@@H	3,4-Dicaffeoylquinic acid
P22303	ACHE	Acetylcholi	[H]₩C(=C	3,5-Di-O-caffeoylquinic acid
P22303	ACHE	Acetylcholi	NC1=CC=	4-Aminobenzoic acid
P22303	ACHE	Acetylcholi	OC1=CC=	4-Hydroxybenzaldehyde
P22303	ACHE	Acetylcholi	CC(C)C1=	5-Isopropyl-2-methylphenol
P22303	ACHE	Acetylcholi	[H]C1=C([	Angelicin
P22303	ACHE	Acetylcholi	OC1=CC=	Apigenin
P22303	ACHE	Acetylcholi	O[C@@H	Aromadendrin
P22303	ACHE	Acetylcholi	OC[C@H]	Astragalin
P22303	ACHE	Acetylcholi	COC1=C2	Bergapten
P22303	ACHE	Acetylcholi	OC1=C2C	Bergaptol
P22303	ACHE	Acetylcholi	COC1=C(	Capsaicin
P22303	ACHE	Acetylcholi	NC(O)=O	Carbamic acid
P22303	ACHE	Acetylcholi	O=C1OC2	coumarin
P22303	ACHE	Acetylcholi	CC(C)=CC	demethylsuberosin
P22303	ACHE	Acetylcholi	O[C@@H	dihydromyricetin
P22303	ACHE	Acetylcholi	N[C@H](	D-Serine
P22303	ACHE	Acetylcholi	OC1=CC(	Galangin
P22303	ACHE	Acetylcholi	OC1=CC=	kaempferol
P22303	ACHE	Acetylcholi	NC(CO)C(	L-Serine
P22303	ACHE	Acetylcholi	OC1=CC2	Luteolin
P22303	ACHE	Acetylcholi	COC1=CC	Melatonin
P22303	ACHE	Acetylcholi	O[C@@H	Neochlorogenic acid
P22303	ACHE	Acetylcholi	OC1=CC(	phloroglucinol
P22303	ACHE	Acetylcholi	O=C1OC2	Psoralen
P22303	ACHE	Acetylcholi	OC1=CC2	Quercetin
P22303	ACHE	Acetylcholi	OC(=O)C1	Salicylic acid
P22303	ACHE	Acetylcholi	COC1=C(	Scopoletin
P22303	ACHE	Acetylcholi	CC1=CC=	Toluene
P22303	ACHE	Acetylcholi	C[N+]1=C	Trigonelline
P22303	ACHE	Acetylcholi	COC1=C2	Xanthotoxin
P22303	ACHE	Acetylcholi	[H]OC1=C	Xanthotoxol
P05067	APP	Amyloid-b	CC(C)(O)C	(+)-marmesin
P05067	APP	Amyloid-b	O[C@@H	(+)-taxifolin
P05067	APP	Amyloid-b	[H][C@]1(	(S)-Naringenin

P05067	APP	Amyloid-b	COC1=C((	(S)-Reticuline
P05067	APP	Amyloid-b	CC1=CC=	1,2-Dimethylbenzene
P05067	APP	Amyloid-b	CC1=CC((	1,3-Dimethylbenzene
P05067	APP	Amyloid-b	CC1=CC=	1,4-Dimethylbenzene
P05067	APP	Amyloid-b	O[C@@H	3,4-Dicaffeoylquinic acid
P05067	APP	Amyloid-b	[H]₩C(=C	3,5-Di-O-caffeoylquinic acid
P05067	APP	Amyloid-b	NC1=CC=	4-Aminobenzoic acid
P05067	APP	Amyloid-b	OC1=CC=	4-Hydroxybenzaldehyde
P05067	APP	Amyloid-b	CC(C)C1=	5-Isopropyl-2-methylphenol
P05067	APP	Amyloid-b	[H]C1=C([	Angelicin
P05067	APP	Amyloid-b	OC1=CC=	Apigenin
P05067	APP	Amyloid-b	O[C@@H	Aromadendrin
P05067	APP	Amyloid-b	OC[C@H]	Astragalin
P05067	APP	Amyloid-b	COC1=C2	Bergapten
P05067	APP	Amyloid-b	OC1=C2C	Bergaptol
P05067	APP	Amyloid-b	COC1=C((	Capsaicin
P05067	APP	Amyloid-b	NC(O)=O	Carbamic acid
P05067	APP	Amyloid-b	O=C1OC2	coumarin
P05067	APP	Amyloid-b	CC(C)=CC	demethylsuberosin
P05067	APP	Amyloid-b	O[C@@H	dihydromyricetin
P05067	APP	Amyloid-b	N[C@H]((	D-Serine
P05067	APP	Amyloid-b	OC1=CC((	Galangin
P05067	APP	Amyloid-b	OC1=CC=	kaempferol
P05067	APP	Amyloid-b	NC(CO)C(	L-Serine
P05067	APP	Amyloid-b	OC1=CC2	Luteolin
P05067	APP	Amyloid-b	COC1=CC	Melatonin
P05067	APP	Amyloid-b	O[C@@H	Neochlorogenic acid
P05067	APP	Amyloid-b	OC1=CC((	phloroglucinol
P05067	APP	Amyloid-b	O=C1OC2	Psoralen
P05067	APP	Amyloid-b	OC1=CC2	Quercetin
P05067	APP	Amyloid-b	OC(=O)C1	Salicylic acid
P05067	APP	Amyloid-b	COC1=C((	Scopoletin
P05067	APP	Amyloid-b	CC1=CC=	Toluene
P05067	APP	Amyloid-b	C[N+]1=C	Trigonelline
P05067	APP	Amyloid-b	COC1=C2	Xanthotoxin
P05067	APP	Amyloid-b	[H]OC1=C	Xanthotoxol
P56817	BACE1	Beta-secre	CC(C)(O)C	(+)-marmesin
P56817	BACE1	Beta-secre	O[C@@H	(+)-taxifolin
P56817	BACE1	Beta-secre	[H][C@]1(	(S)-Naringenin
P56817	BACE1	Beta-secre	COC1=C((	(S)-Reticuline
P56817	BACE1	Beta-secre	CC1=CC=	1,2-Dimethylbenzene
P56817	BACE1	Beta-secre	CC1=CC((	1,3-Dimethylbenzene
P56817	BACE1	Beta-secre	CC1=CC=	1,4-Dimethylbenzene
P56817	BACE1	Beta-secre	O[C@@H	3,4-Dicaffeoylquinic acid

P56817	BACE1	Beta-secre	[H]₩C(=C	3,5-Di-O-caffeoylquinic acid
P56817	BACE1	Beta-secre	NC1=CC=	4-Aminobenzoic acid
P56817	BACE1	Beta-secre	OC1=CC=	4-Hydroxybenzaldehyde
P56817	BACE1	Beta-secre	CC(C)C1=	5-Isopropyl-2-methylphenol
P56817	BACE1	Beta-secre	[H]C1=C([	Angelicin
P56817	BACE1	Beta-secre	OC1=CC=	Apigenin
P56817	BACE1	Beta-secre	O[C@@H	Aromadendrin
P56817	BACE1	Beta-secre	OC[C@H]	Astragalin
P56817	BACE1	Beta-secre	COC1=C2	Bergapten
P56817	BACE1	Beta-secre	OC1=C2C	Bergaptol
P56817	BACE1	Beta-secre	COC1=C(	Capsaicin
P56817	BACE1	Beta-secre	NC(O)=O	Carbamic acid
P56817	BACE1	Beta-secre	O=C1OC2	coumarin
P56817	BACE1	Beta-secre	CC(C)=CC	demethylsuberosin
P56817	BACE1	Beta-secre	O[C@@H	dihydromyricetin
P56817	BACE1	Beta-secre	N[C@H](	D-Serine
P56817	BACE1	Beta-secre	OC1=CC(	Galangin
P56817	BACE1	Beta-secre	OC1=CC=	kaempferol
P56817	BACE1	Beta-secre	NC(CO)C(	L-Serine
P56817	BACE1	Beta-secre	OC1=CC2	Luteolin
P56817	BACE1	Beta-secre	COC1=CC	Melatonin
P56817	BACE1	Beta-secre	O[C@@H	Neochlorogenic acid
P56817	BACE1	Beta-secre	OC1=CC(	phloroglucinol
P56817	BACE1	Beta-secre	O=C1OC2	Psoralen
P56817	BACE1	Beta-secre	OC1=CC2	Quercetin
P56817	BACE1	Beta-secre	OC(=O)C1	Salicylic acid
P56817	BACE1	Beta-secre	COC1=C(	Scopoletin
P56817	BACE1	Beta-secre	CC1=CC=	Toluene
P56817	BACE1	Beta-secre	C[N+]1=C	Trigonelline
P56817	BACE1	Beta-secre	COC1=C2	Xanthotoxin
P56817	BACE1	Beta-secre	[H]OC1=C	Xanthotoxol
P10636	MAPT	Microtubu	CC(C)(O)C	(+)-marmesin
P10636	MAPT	Microtubu	O[C@@H	(+)-taxifolin
P10636	MAPT	Microtubu	[H][C@]1(	(S)-Naringenin
P10636	MAPT	Microtubu	COC1=C(	(S)-Reticuline
P10636	MAPT	Microtubu	CC1=CC=	1,2-Dimethylbenzene
P10636	MAPT	Microtubu	CC1=CC(	1,3-Dimethylbenzene
P10636	MAPT	Microtubu	CC1=CC=	1,4-Dimethylbenzene
P10636	MAPT	Microtubu	O[C@@H	3,4-Dicaffeoylquinic acid
P10636	MAPT	Microtubu	[H]₩C(=C	3,5-Di-O-caffeoylquinic acid
P10636	MAPT	Microtubu	NC1=CC=	4-Aminobenzoic acid
P10636	MAPT	Microtubu	OC1=CC=	4-Hydroxybenzaldehyde
P10636	MAPT	Microtubu	CC(C)C1=	5-Isopropyl-2-methylphenol
P10636	MAPT	Microtubu	[H]C1=C([	Angelicin

P10636	MAPT	Microtubu	OC1=CC=	Apigenin
P10636	MAPT	Microtubu	O[C@@H	Aromadendrin
P10636	MAPT	Microtubu	OC[C@H]	Astragalin
P10636	MAPT	Microtubu	COC1=C2	Bergapten
P10636	MAPT	Microtubu	OC1=C2C	Bergaptol
P10636	MAPT	Microtubu	COC1=C((	Capsaicin
P10636	MAPT	Microtubu	NC(O)=O	Carbamic acid
P10636	MAPT	Microtubu	O=C1OC2	coumarin
P10636	MAPT	Microtubu	CC(C)=CC	demethylsuberosin
P10636	MAPT	Microtubu	O[C@@H	dihydromyricetin
P10636	MAPT	Microtubu	N[C@H]((	D-Serine
P10636	MAPT	Microtubu	OC1=CC((	Galangin
P10636	MAPT	Microtubu	OC1=CC=	kaempferol
P10636	MAPT	Microtubu	NC(CO)C(	L-Serine
P10636	MAPT	Microtubu	OC1=CC2	Luteolin
P10636	MAPT	Microtubu	COC1=CC	Melatonin
P10636	MAPT	Microtubu	O[C@@H	Neochlorogenic acid
P10636	MAPT	Microtubu	OC1=CC((	phloroglucinol
P10636	MAPT	Microtubu	O=C1OC2	Psoralen
P10636	MAPT	Microtubu	OC1=CC2	Quercetin
P10636	MAPT	Microtubu	OC(=O)C1	Salicylic acid
P10636	MAPT	Microtubu	COC1=C((	Scopoletin
P10636	MAPT	Microtubu	CC1=CC=	Toluene
P10636	MAPT	Microtubu	C[N+]1=C	Trigonelline
P10636	MAPT	Microtubu	COC1=C2	Xanthotoxin
P10636	MAPT	Microtubu	[H]OC1=C	Xanthotoxol
P49768	PSEN1	Presenilin-	CC(C)(O)C	(+)-marmesin
P49768	PSEN1	Presenilin-	O[C@@H	(+)-taxifolin
P49768	PSEN1	Presenilin-	[H][C@]1(	(S)-Naringenin
P49768	PSEN1	Presenilin-	COC1=C((	(S)-Reticuline
P49768	PSEN1	Presenilin-	CC1=CC=	1,2-Dimethylbenzene
P49768	PSEN1	Presenilin-	CC1=CC((	1,3-Dimethylbenzene
P49768	PSEN1	Presenilin-	CC1=CC=	1,4-Dimethylbenzene
P49768	PSEN1	Presenilin-	O[C@@H	3,4-Dicaffeoylquinic acid
P49768	PSEN1	Presenilin-	[H]₩C(=C	3,5-Di-O-caffeoylquinic acid
P49768	PSEN1	Presenilin-	NC1=CC=	4-Aminobenzoic acid
P49768	PSEN1	Presenilin-	OC1=CC=	4-Hydroxybenzaldehyde
P49768	PSEN1	Presenilin-	CC(C)C1=	5-Isopropyl-2-methylphenol
P49768	PSEN1	Presenilin-	[H]C1=C([	Angelicin
P49768	PSEN1	Presenilin-	OC1=CC=	Apigenin
P49768	PSEN1	Presenilin-	O[C@@H	Aromadendrin
P49768	PSEN1	Presenilin-	OC[C@H]	Astragalin
P49768	PSEN1	Presenilin-	COC1=C2	Bergapten
P49768	PSEN1	Presenilin-	OC1=C2C	Bergaptol

P49768	PSEN1	Presenilin-	COC1=C((	Capsaicin
P49768	PSEN1	Presenilin-	NC(O)=O	Carbamic acid
P49768	PSEN1	Presenilin-	O=C1OC2	coumarin
P49768	PSEN1	Presenilin-	CC(C)=CC	demethylsuberosin
P49768	PSEN1	Presenilin-	O[C@@H	dihydromyricetin
P49768	PSEN1	Presenilin-	N[C@H]((	D-Serine
P49768	PSEN1	Presenilin-	OC1=CC((	Galangin
P49768	PSEN1	Presenilin-	OC1=CC=	kaempferol
P49768	PSEN1	Presenilin-	NC(CO)C(	L-Serine
P49768	PSEN1	Presenilin-	OC1=CC2	Luteolin
P49768	PSEN1	Presenilin-	COC1=CC	Melatonin
P49768	PSEN1	Presenilin-	O[C@@H	Neochlorogenic acid
P49768	PSEN1	Presenilin-	OC1=CC((	phloroglucinol
P49768	PSEN1	Presenilin-	O=C1OC2	Psoralen
P49768	PSEN1	Presenilin-	OC1=CC2	Quercetin
P49768	PSEN1	Presenilin-	OC(=O)C1	Salicylic acid
P49768	PSEN1	Presenilin-	COC1=C((	Scopoletin
P49768	PSEN1	Presenilin-	CC1=CC=	Toluene
P49768	PSEN1	Presenilin-	C[N+]1=C	Trigonelline
P49768	PSEN1	Presenilin-	COC1=C2	Xanthotoxin
P49768	PSEN1	Presenilin-	[H]OC1=C	Xanthotoxol
P01375	TNF	Tumor nec	CC(C)(O)C	(+)-marmesin
P01375	TNF	Tumor nec	O[C@@H	(+)-taxifolin
P01375	TNF	Tumor nec	[H][C@]1(	(S)-Naringenin
P01375	TNF	Tumor nec	COC1=C((	(S)-Reticuline
P01375	TNF	Tumor nec	CC1=CC=	1,2-Dimethylbenzene
P01375	TNF	Tumor nec	CC1=CC((	1,3-Dimethylbenzene
P01375	TNF	Tumor nec	CC1=CC=	1,4-Dimethylbenzene
P01375	TNF	Tumor nec	O[C@@H	3,4-Dicaffeoylquinic acid
P01375	TNF	Tumor nec	[H]₩C(=C	3,5-Di-O-caffeoylquinic acid
P01375	TNF	Tumor nec	NC1=CC=	4-Aminobenzoic acid
P01375	TNF	Tumor nec	OC1=CC=	4-Hydroxybenzaldehyde
P01375	TNF	Tumor nec	CC(C)C1=	5-Isopropyl-2-methylphenol
P01375	TNF	Tumor nec	[H]C1=C([	Angelicin
P01375	TNF	Tumor nec	OC1=CC=	Apigenin
P01375	TNF	Tumor nec	O[C@@H	Aromadendrin
P01375	TNF	Tumor nec	OC[C@H]	Astragalin
P01375	TNF	Tumor nec	COC1=C2	Bergapten
P01375	TNF	Tumor nec	OC1=C2C	Bergaptol
P01375	TNF	Tumor nec	COC1=C((	Capsaicin
P01375	TNF	Tumor nec	NC(O)=O	Carbamic acid
P01375	TNF	Tumor nec	O=C1OC2	coumarin
P01375	TNF	Tumor nec	CC(C)=CC	demethylsuberosin
P01375	TNF	Tumor nec	O[C@@H	dihydromyricetin

P01375	TNF	Tumor nec	N[C@H]((	D-Serine
P01375	TNF	Tumor nec	OC1=CC((	Galangin
P01375	TNF	Tumor nec	OC1=CC=	kaempferol
P01375	TNF	Tumor nec	NC(CO)C(	L-Serine
P01375	TNF	Tumor nec	OC1=CC2	Luteolin
P01375	TNF	Tumor nec	COC1=CC	Melatonin
P01375	TNF	Tumor nec	O[C@@H	Neochlorogenic acid
P01375	TNF	Tumor nec	OC1=CC((	phloroglucinol
P01375	TNF	Tumor nec	O=C1OC2	Psoralen
P01375	TNF	Tumor nec	OC1=CC2	Quercetin
P01375	TNF	Tumor nec	OC(=O)C1	Salicylic acid
P01375	TNF	Tumor nec	COC1=C((	Scopoletin
P01375	TNF	Tumor nec	CC1=CC=	Toluene
P01375	TNF	Tumor nec	C[N+]1=C	Trigonelline
P01375	TNF	Tumor nec	COC1=C2	Xanthotoxin
P01375	TNF	Tumor nec	[H]OC1=C	Xanthotoxol
P55072	VCP	Valosin Co	CC(C)(O)C	(+)-marmesin
P55072	VCP	Valosin Co	O[C@@H	(+)-taxifolin
P55072	VCP	Valosin Co	[H][C@]1(	(S)-Naringenin
P55072	VCP	Valosin Co	COC1=C((	(S)-Reticuline
P55072	VCP	Valosin Co	CC1=CC=	1,2-Dimethylbenzene
P55072	VCP	Valosin Co	CC1=CC((	1,3-Dimethylbenzene
P55072	VCP	Valosin Co	CC1=CC=	1,4-Dimethylbenzene
P55072	VCP	Valosin Co	O[C@@H	3,4-Dicaffeoylquinic acid
P55072	VCP	Valosin Co	[H]₩C(=C	3,5-Di-O-caffeoylquinic acid
P55072	VCP	Valosin Co	NC1=CC=	4-Aminobenzoic acid
P55072	VCP	Valosin Co	OC1=CC=	4-Hydroxybenzaldehyde
P55072	VCP	Valosin Co	CC(C)C1=	5-Isopropyl-2-methylphenol
P55072	VCP	Valosin Co	[H]C1=C([	Angelicin
P55072	VCP	Valosin Co	OC1=CC=	Apigenin
P55072	VCP	Valosin Co	O[C@@H	Aromadendrin
P55072	VCP	Valosin Co	OC[C@H]	Astragalin
P55072	VCP	Valosin Co	COC1=C2	Bergapten
P55072	VCP	Valosin Co	OC1=C2C	Bergaptol
P55072	VCP	Valosin Co	COC1=C((	Capsaicin
P55072	VCP	Valosin Co	NC(O)=O	Carbamic acid
P55072	VCP	Valosin Co	O=C1OC2	coumarin
P55072	VCP	Valosin Co	CC(C)=CC	demethylsuberosin
P55072	VCP	Valosin Co	O[C@@H	dihydromyricetin
P55072	VCP	Valosin Co	N[C@H]((	D-Serine
P55072	VCP	Valosin Co	OC1=CC((	Galangin
P55072	VCP	Valosin Co	OC1=CC=	kaempferol
P55072	VCP	Valosin Co	NC(CO)C(	L-Serine
P55072	VCP	Valosin Co	OC1=CC2	Luteolin

P55072	VCP	Valosin Co	COC1=CC	Melatonin
P55072	VCP	Valosin Co	O[C@@H	Neochlorogenic acid
P55072	VCP	Valosin Co	OC1=CC(	phloroglucinol
P55072	VCP	Valosin Co	O=C1OC2	Psoralen
P55072	VCP	Valosin Co	OC1=CC2	Quercetin
P55072	VCP	Valosin Co	OC(=O)C1	Salicylic acid
P55072	VCP	Valosin Co	COC1=C(	Scopoletin
P55072	VCP	Valosin Co	CC1=CC=	Toluene
P55072	VCP	Valosin Co	C[N+]1=C	Trigonelline
P55072	VCP	Valosin Co	COC1=C2	Xanthotoxin
P55072	VCP	Valosin Co	[H]OC1=C	Xanthotoxol

Spplementary Table S3. Docking information between Alzheimer's disease related protein and natural compounds

ID_Uniprot	Protein	Descriptior	SMILES	Chemical N	Docking Score_Binding Energy(kcal/mol)
P22303	ACHE	Acetylcholi	CC(C)(O)C	(+)-marme	-8.63519
P22303	ACHE	Acetylcholi	O[C@@H	(+)-taxifoli	-9.25852
P22303	ACHE	Acetylcholi	[H][C@]1(	(S)-Naring	-10.3807
P22303	ACHE	Acetylcholi	COC1=C((	(S)-Reticuli	-10.8014
P22303	ACHE	Acetylcholi	CC1=CC=	1,2-Dimetl	-6.26273
P22303	ACHE	Acetylcholi	CC1=CC((	1,3-Dimetl	-6.24712
P22303	ACHE	Acetylcholi	CC1=CC=	1,4-Dimetl	-5.64227
P22303	ACHE	Acetylcholi	O[C@@H	3,4-Dicaffe	-10.1511
P22303	ACHE	Acetylcholi	[H]₩C(=C	3,5-Di-O-c	-9.93811
P22303	ACHE	Acetylcholi	NC1=CC=	4-Aminobe	-6.35319
P22303	ACHE	Acetylcholi	OC1=CC=	4-Hydroxyl	-5.97149
P22303	ACHE	Acetylcholi	CC(C)C1=	5-Isopropy	-7.17423
P22303	ACHE	Acetylcholi	[H]C1=C([	Angelicin	-8.20834
P22303	ACHE	Acetylcholi	OC1=CC=	Apigenin	-9.46808
P22303	ACHE	Acetylcholi	O[C@@H	Aromaden	-9.08877
P22303	ACHE	Acetylcholi	OC[C@H]	Astragalin	-10.1251
P22303	ACHE	Acetylcholi	COC1=C2	Bergapten	-8.78266
P22303	ACHE	Acetylcholi	OC1=C2C	Bergaptol	-8.17088
P22303	ACHE	Acetylcholi	COC1=C((	Capsaicin	-11.6749
P22303	ACHE	Acetylcholi	NC(O)=O	Carbamic a	-5.25888
P22303	ACHE	Acetylcholi	O=C1OC2	coumarin	-7.85157
P22303	ACHE	Acetylcholi	CC(C)=CC	demethylsı	-9.18605
P22303	ACHE	Acetylcholi	O[C@@H	dihydromy	-9.64439
P22303	ACHE	Acetylcholi	N[C@H]((	D-Serine	-6.62597
P22303	ACHE	Acetylcholi	OC1=CC((	Galangin	-9.5971
P22303	ACHE	Acetylcholi	OC1=CC=	kaempfero	-9.99062
P22303	ACHE	Acetylcholi	NC(CO)C(	L-Serine	-6.4754
P22303	ACHE	Acetylcholi	OC1=CC2	Luteolin	-10.8172
P22303	ACHE	Acetylcholi	COC1=CC	Melatonin	-9.13937
P22303	ACHE	Acetylcholi	O[C@@H	Neochloro	-8.11787
P22303	ACHE	Acetylcholi	OC1=CC((	phlorogluc	-7.0478
P22303	ACHE	Acetylcholi	O=C1OC2	Psoralen	-9.74874
P22303	ACHE	Acetylcholi	OC1=CC2	Quercetin	-9.87744
P22303	ACHE	Acetylcholi	OC(=O)C1	Salicylic ac	-5.50899
P22303	ACHE	Acetylcholi	COC1=C((	Scopoletin	-8.61238
P22303	ACHE	Acetylcholi	CC1=CC=	Toluene	-5.93509
P22303	ACHE	Acetylcholi	C[N+]1=C	Trigonellin	-6.66333
P22303	ACHE	Acetylcholi	COC1=C2	Xanthotoxi	-8.02819
P22303	ACHE	Acetylcholi	[H]OC1=C	Xanthotox	-7.97561
P05067	APP	Amyloid-b	CC(C)(O)C	(+)-marme	-7.88164
P05067	APP	Amyloid-b	O[C@@H	(+)-taxifoli	-7.83485
P05067	APP	Amyloid-b	[H][C@]1(	(S)-Naring	-8.08565
P05067	APP	Amyloid-b	COC1=C((	(S)-Reticuli	-9.27369
P05067	APP	Amyloid-b	CC1=CC=	1,2-Dimetl	-4.88808
P05067	APP	Amyloid-b	CC1=CC((	1,3-Dimetl	-4.93969
P05067	APP	Amyloid-b	CC1=CC=	1,4-Dimetl	-4.94863
P05067	APP	Amyloid-b	O[C@@H	3,4-Dicaffe	-9.09332
P05067	APP	Amyloid-b	[H]₩C(=C	3,5-Di-O-c	-10.3419
P05067	APP	Amyloid-b	NC1=CC=	4-Aminobe	-5.935
P05067	APP	Amyloid-b	OC1=CC=	4-Hydroxyl	-4.67787
P05067	APP	Amyloid-b	CC(C)C1=	5-Isopropy	-5.51826
P05067	APP	Amyloid-b	[H]C1=C([	Angelicin	-8.19427

P05067	APP	Amyloid-b	OC1=CC=	Apigenin	-7.31561
P05067	APP	Amyloid-b	O[C@@H	Aromaden	-7.81537
P05067	APP	Amyloid-b	OC[C@H]	Astragalin	-7.91162
P05067	APP	Amyloid-b	COC1=C2	Bergapten	-8.42036
P05067	APP	Amyloid-b	OC1=C2C	Bergaptol	-6.03549
P05067	APP	Amyloid-b	COC1=C((	Capsaicin	-7.98599
P05067	APP	Amyloid-b	NC(O)=O	Carbamic a	-4.16434
P05067	APP	Amyloid-b	O=C1OC2	coumarin	-8.34463
P05067	APP	Amyloid-b	CC(C)=CC	demethylsu	-7.99756
P05067	APP	Amyloid-b	O[C@@H	dihydromy	-7.95324
P05067	APP	Amyloid-b	N[C@H](C	D-Serine	-4.21172
P05067	APP	Amyloid-b	OC1=CC((	Galangin	-7.87404
P05067	APP	Amyloid-b	OC1=CC=	kaempfero	-7.70778
P05067	APP	Amyloid-b	NC(CO)C(	L-Serine	-5.40772
P05067	APP	Amyloid-b	OC1=CC2	Luteolin	-8.25183
P05067	APP	Amyloid-b	COC1=CC	Melatonin	-7.69848
P05067	APP	Amyloid-b	O[C@@H	Neochloro	-8.19148
P05067	APP	Amyloid-b	OC1=CC((	phlorogluc	-5.4667
P05067	APP	Amyloid-b	O=C1OC2	Psoralen	-7.93181
P05067	APP	Amyloid-b	OC1=CC2	Quercetin	-8.61755
P05067	APP	Amyloid-b	OC(=O)C1	Salicylic ac	-5.03412
P05067	APP	Amyloid-b	COC1=C((	Scopoletin	-8.13629
P05067	APP	Amyloid-b	CC1=CC=	Toluene	-4.74413
P05067	APP	Amyloid-b	C[N+]1=C	Trigonellin	-4.8319
P05067	APP	Amyloid-b	COC1=C2	Xanthotoxi	-8.49542
P05067	APP	Amyloid-b	[H]OC1=C	Xanthotoxo	-7.8464
P56817	BACE1	Beta-secre	CC(C)(O)C	(+)-marme	-8.20841
P56817	BACE1	Beta-secre	O[C@@H	(+)-taxifoli	-8.72505
P56817	BACE1	Beta-secre	[H][C@]1(	(S)-Naringe	-7.83085
P56817	BACE1	Beta-secre	COC1=C((	(S)-Reticuli	-9.46145
P56817	BACE1	Beta-secre	CC1=CC=	1,2-Dimeth	-5.00942
P56817	BACE1	Beta-secre	CC1=CC((	1,3-Dimeth	-5.42495
P56817	BACE1	Beta-secre	CC1=CC=	1,4-Dimeth	-5.42329
P56817	BACE1	Beta-secre	O[C@@H	3,4-Dicaffe	-9.19709
P56817	BACE1	Beta-secre	[H]₩C(=C	3,5-Di-O-c	-9.48865
P56817	BACE1	Beta-secre	NC1=CC=	4-Aminobe	-5.17099
P56817	BACE1	Beta-secre	OC1=CC=	4-Hydroxyl	-5.82632
P56817	BACE1	Beta-secre	CC(C)C1=	5-Isopropy	-6.41147
P56817	BACE1	Beta-secre	[H]C1=C([	Angelicin	-8.6512
P56817	BACE1	Beta-secre	OC1=CC=	Apigenin	-9.06248
P56817	BACE1	Beta-secre	O[C@@H	Aromaden	-8.6641
P56817	BACE1	Beta-secre	OC[C@H]	Astragalin	-8.01321
P56817	BACE1	Beta-secre	COC1=C2	Bergapten	-8.10697
P56817	BACE1	Beta-secre	OC1=C2C	Bergaptol	-7.39488
P56817	BACE1	Beta-secre	COC1=C((	Capsaicin	-8.60264
P56817	BACE1	Beta-secre	NC(O)=O	Carbamic a	-4.88808
P56817	BACE1	Beta-secre	O=C1OC2	coumarin	-8.25965
P56817	BACE1	Beta-secre	CC(C)=CC	demethylsu	-7.75106
P56817	BACE1	Beta-secre	O[C@@H	dihydromy	-8.70795
P56817	BACE1	Beta-secre	N[C@H](C	D-Serine	-5.47727
P56817	BACE1	Beta-secre	OC1=CC((	Galangin	-8.87039
P56817	BACE1	Beta-secre	OC1=CC=	kaempfero	-8.59285
P56817	BACE1	Beta-secre	NC(CO)C(	L-Serine	-5.1189
P56817	BACE1	Beta-secre	OC1=CC2	Luteolin	-8.18569

P56817	BACE1	Beta-secre	COC1=CC	Melatonin	-8.4926
P56817	BACE1	Beta-secre	O[C@@H	Neochloro	-8.74942
P56817	BACE1	Beta-secre	OC1=CC((	phlorogluc	-6.18982
P56817	BACE1	Beta-secre	O=C1OC2	Psoralen	-8.1199
P56817	BACE1	Beta-secre	OC1=CC2	Quercetin	-8.5642
P56817	BACE1	Beta-secre	OC(=O)C1	Salicylic ac	-5.73591
P56817	BACE1	Beta-secre	COC1=C((	Scopoletin	-8.29791
P56817	BACE1	Beta-secre	CC1=CC=	Toluene	-4.82804
P56817	BACE1	Beta-secre	C[N+]1=C	Trigonellin	-5.75723
P56817	BACE1	Beta-secre	COC1=C2	Xanthotoxi	-8.74035
P56817	BACE1	Beta-secre	[H]OC1=C	Xanthotox	-8.12889
P10636	MAPT	Microtubul	CC(C)(O)C	(+)-marme	-9.68333
P10636	MAPT	Microtubul	O[C@@H	(+)-taxifoli	-9.41533
P10636	MAPT	Microtubul	[H][C@]1(	(S)-Naring	-8.97828
P10636	MAPT	Microtubul	COC1=C((	(S)-Reticuli	-10.9793
P10636	MAPT	Microtubul	CC1=CC=	1,2-Dimetł	-6.37406
P10636	MAPT	Microtubul	CC1=CC((	1,3-Dimetł	-6.6314
P10636	MAPT	Microtubul	CC1=CC=	1,4-Dimetł	-6.51537
P10636	MAPT	Microtubul	O[C@@H	3,4-Dicaffe	-11.5073
P10636	MAPT	Microtubul	[H]₩C(=C	3,5-Di-O-c	-11.1635
P10636	MAPT	Microtubul	NC1=CC=	4-Aminobe	-5.72974
P10636	MAPT	Microtubul	OC1=CC=	4-Hydroxyl	-5.64936
P10636	MAPT	Microtubul	CC(C)C1=	5-Isopropy	-8.00175
P10636	MAPT	Microtubul	[H]C1=C([	Angelicin	-8.63489
P10636	MAPT	Microtubul	OC1=CC=	Apigenin	-8.96955
P10636	MAPT	Microtubul	O[C@@H	Aromaden	-9.33306
P10636	MAPT	Microtubul	OC[C@H]	Astragalin	-9.42236
P10636	MAPT	Microtubul	COC1=C2	Bergapten	-8.12474
P10636	MAPT	Microtubul	OC1=C2C	Bergaptol	-7.96617
P10636	MAPT	Microtubul	COC1=C((	Capsaicin	-10.7349
P10636	MAPT	Microtubul	NC(O)=O	Carbamic a	-4.28102
P10636	MAPT	Microtubul	O=C1OC2	coumarin	-8.04618
P10636	MAPT	Microtubul	CC(C)=CC	demethyls	-10.3466
P10636	MAPT	Microtubul	O[C@@H	dihydromy	-9.03025
P10636	MAPT	Microtubul	N[C@H](	D-Serine	-4.51769
P10636	MAPT	Microtubul	OC1=CC((	Galangin	-10.0342
P10636	MAPT	Microtubul	OC1=CC=	kaempfero	-9.73013
P10636	MAPT	Microtubul	NC(CO)C(	L-Serine	-4.84361
P10636	MAPT	Microtubul	OC1=CC2	Luteolin	-9.32804
P10636	MAPT	Microtubul	COC1=CC	Melatonin	-8.84982
P10636	MAPT	Microtubul	O[C@@H	Neochloro	-9.48405
P10636	MAPT	Microtubul	OC1=CC((	phlorogluc	-6.41147
P10636	MAPT	Microtubul	O=C1OC2	Psoralen	-8.76102
P10636	MAPT	Microtubul	OC1=CC2	Quercetin	-10.306
P10636	MAPT	Microtubul	OC(=O)C1	Salicylic ac	-6.46326
P10636	MAPT	Microtubul	COC1=C((	Scopoletin	-8.79341
P10636	MAPT	Microtubul	CC1=CC=	Toluene	-5.90985
P10636	MAPT	Microtubul	C[N+]1=C	Trigonellin	-5.57248
P10636	MAPT	Microtubul	COC1=C2	Xanthotoxi	-8.64307
P10636	MAPT	Microtubul	[H]OC1=C	Xanthotox	-8.37387
P49768	PSEN1	Presenilin-	CC(C)(O)C	(+)-marme	-8.65351
P49768	PSEN1	Presenilin-	O[C@@H	(+)-taxifoli	-8.40763
P49768	PSEN1	Presenilin-	[H][C@]1(	(S)-Naring	-8.46736
P49768	PSEN1	Presenilin-	COC1=C((	(S)-Reticuli	-9.92719

P49768	PSEN1	Presenilin-	CC1=CC=	1,2-Dimetl	-5.6063
P49768	PSEN1	Presenilin-	CC1=CC((	1,3-Dimetl	-5.95278
P49768	PSEN1	Presenilin-	CC1=CC=	1,4-Dimetl	-4.84361
P49768	PSEN1	Presenilin-	O[C@@H	3,4-Dicaffe	-8.32756
P49768	PSEN1	Presenilin-	[H]₩C(=C	3,5-Di-O-c	-8.88441
P49768	PSEN1	Presenilin-	NC1=CC=	4-Aminobe	-5.08067
P49768	PSEN1	Presenilin-	OC1=CC=	4-Hydroxyl	-5.87802
P49768	PSEN1	Presenilin-	CC(C)C1=	5-Isopropy	-7.44107
P49768	PSEN1	Presenilin-	[H]C1=C([	Angelicin	-8.3602
P49768	PSEN1	Presenilin-	OC1=CC=	Apigenin	-8.87039
P49768	PSEN1	Presenilin-	O[C@@H	Aromaden	-8.27089
P49768	PSEN1	Presenilin-	OC[C@H]	Astragalin	-9.77993
P49768	PSEN1	Presenilin-	COC1=C2	Bergapten	-8.7882
P49768	PSEN1	Presenilin-	OC1=C2C	Bergaptol	-7.3493
P49768	PSEN1	Presenilin-	COC1=C((	Capsaicin	-8.08323
P49768	PSEN1	Presenilin-	NC(O)=O	Carbamic a	-4.50997
P49768	PSEN1	Presenilin-	O=C1OC2	coumarin	-8.094
P49768	PSEN1	Presenilin-	CC(C)=CC	demethylsı	-8.5642
P49768	PSEN1	Presenilin-	O[C@@H	dihydromy	-8.76043
P49768	PSEN1	Presenilin-	N[C@H]((	D-Serine	-5.30809
P49768	PSEN1	Presenilin-	OC1=CC((	Galangin	-7.50687
P49768	PSEN1	Presenilin-	OC1=CC=	kaempfero	-9.20027
P49768	PSEN1	Presenilin-	NC(CO)C(	L-Serine	-5.05447
P49768	PSEN1	Presenilin-	OC1=CC2	Luteolin	-7.78648
P49768	PSEN1	Presenilin-	COC1=CC	Melatonin	-8.70233
P49768	PSEN1	Presenilin-	O[C@@H	Neochloro	-8.85662
P49768	PSEN1	Presenilin-	OC1=CC((	phlorogluc	-5.69026
P49768	PSEN1	Presenilin-	O=C1OC2	Psoralen	-8.77134
P49768	PSEN1	Presenilin-	OC1=CC2	Quercetin	-7.61804
P49768	PSEN1	Presenilin-	OC(=O)C1	Salicylic ac	-6.06514
P49768	PSEN1	Presenilin-	COC1=C((	Scopoletin	-8.33372
P49768	PSEN1	Presenilin-	CC1=CC=	Toluene	-5.51174
P49768	PSEN1	Presenilin-	C[N+]1=C	Trigonellin	-6.1965
P49768	PSEN1	Presenilin-	COC1=C2	Xanthotoxi	-8.59207
P49768	PSEN1	Presenilin-	[H]OC1=C	Xanthotox	-8.1854
P01375	TNF	Tumor nec	CC(C)(O)C	(+)-marme	-11.2804
P01375	TNF	Tumor nec	O[C@@H	(+)-taxifoli	-9.70131
P01375	TNF	Tumor nec	[H][C@]1(	(S)-Naring	-10.4774
P01375	TNF	Tumor nec	COC1=C((	(S)-Reticuli	-13.2511
P01375	TNF	Tumor nec	CC1=CC=	1,2-Dimetl	-5.84034
P01375	TNF	Tumor nec	CC1=CC((	1,3-Dimetl	-5.88249
P01375	TNF	Tumor nec	CC1=CC=	1,4-Dimetl	-8.30372
P01375	TNF	Tumor nec	O[C@@H	3,4-Dicaffe	-12.0675
P01375	TNF	Tumor nec	[H]₩C(=C	3,5-Di-O-c	-12.2422
P01375	TNF	Tumor nec	NC1=CC=	4-Aminobe	-8.28713
P01375	TNF	Tumor nec	OC1=CC=	4-Hydroxyl	-5.05447
P01375	TNF	Tumor nec	CC(C)C1=	5-Isopropy	-7.39279
P01375	TNF	Tumor nec	[H]C1=C([	Angelicin	-9.09982
P01375	TNF	Tumor nec	OC1=CC=	Apigenin	-11.0301
P01375	TNF	Tumor nec	O[C@@H	Aromaden	-10.3654
P01375	TNF	Tumor nec	OC[C@H]	Astragalin	-9.35933
P01375	TNF	Tumor nec	COC1=C2	Bergapten	-10.8915
P01375	TNF	Tumor nec	OC1=C2C	Bergaptol	-10.5829
P01375	TNF	Tumor nec	COC1=C((	Capsaicin	-12.2162

P01375	TNF	Tumor nec	NC(O)=O	Carbamic a	-4.35081
P01375	TNF	Tumor nec	O=C1OC2	coumarin	-8.74123
P01375	TNF	Tumor nec	CC(C)=CC	demethylsu	-11.5875
P01375	TNF	Tumor nec	O[C@@H	dihydromy	-9.48865
P01375	TNF	Tumor nec	N[C@H](C	D-Serine	-5.35214
P01375	TNF	Tumor nec	OC1=CC((	Galangin	-10.7804
P01375	TNF	Tumor nec	OC1=CC=	kaempfero	-10.8878
P01375	TNF	Tumor nec	NC(CO)C(	L-Serine	-5.35214
P01375	TNF	Tumor nec	OC1=CC2	Luteolin	-10.9416
P01375	TNF	Tumor nec	COC1=CC	Melatonin	-11.3117
P01375	TNF	Tumor nec	O[C@@H	Neochloro	-9.85662
P01375	TNF	Tumor nec	OC1=CC((	phlorogluc	-5.52389
P01375	TNF	Tumor nec	O=C1OC2	Psoralen	-10.1993
P01375	TNF	Tumor nec	OC1=CC2	Quercetin	-9.66945
P01375	TNF	Tumor nec	OC(=O)C1	Salicylic ac	-6.02108
P01375	TNF	Tumor nec	COC1=C((	Scopoletin	-8.49968
P01375	TNF	Tumor nec	CC1=CC=	Toluene	-7.69387
P01375	TNF	Tumor nec	C[N+]1=C	Trigonellin	-7.10697
P01375	TNF	Tumor nec	COC1=C2	Xanthotoxi	-9.57647
P01375	TNF	Tumor nec	[H]OC1=C	Xanthotox	-9.0607
P55072	VCP	Valosin Co	CC(C)(O)C	(+)-marme	-8.88854
P55072	VCP	Valosin Co	O[C@@H	(+)-taxifoli	-9.18973
P55072	VCP	Valosin Co	[H][C@]1(	(S)-Naring	-8.3642
P55072	VCP	Valosin Co	COC1=C((	(S)-Reticuli	-10.207
P55072	VCP	Valosin Co	CC1=CC=	1,2-Dimeth	-5.86916
P55072	VCP	Valosin Co	CC1=CC((	1,3-Dimeth	-5.90369
P55072	VCP	Valosin Co	CC1=CC=	1,4-Dimeth	-6.10784
P55072	VCP	Valosin Co	O[C@@H	3,4-Dicaffe	-10.44
P55072	VCP	Valosin Co	[H]₩C(=C	3,5-Di-O-c	-10.5284
P55072	VCP	Valosin Co	NC1=CC=	4-Aminobe	-6.10984
P55072	VCP	Valosin Co	OC1=CC=	4-Hydroxyl	-6.60073
P55072	VCP	Valosin Co	CC(C)C1=	5-Isopropy	-6.64322
P55072	VCP	Valosin Co	[H]C1=C([	Angelicin	-8.23323
P55072	VCP	Valosin Co	OC1=CC=	Apigenin	-9.19498
P55072	VCP	Valosin Co	O[C@@H	Aromaden	-8.14386
P55072	VCP	Valosin Co	OC[C@H]	Astragalin	-9.62257
P55072	VCP	Valosin Co	COC1=C2	Bergapten	-8.90758
P55072	VCP	Valosin Co	OC1=C2C	Bergaptol	-8.3887
P55072	VCP	Valosin Co	COC1=C((	Capsaicin	-9.02261
P55072	VCP	Valosin Co	NC(O)=O	Carbamic a	-4.82804
P55072	VCP	Valosin Co	O=C1OC2	coumarin	-8.24025
P55072	VCP	Valosin Co	CC(C)=CC	demethylsu	-8.03807
P55072	VCP	Valosin Co	O[C@@H	dihydromy	-9.3095
P55072	VCP	Valosin Co	N[C@H](C	D-Serine	-5.85174
P55072	VCP	Valosin Co	OC1=CC((	Galangin	-8.55953
P55072	VCP	Valosin Co	OC1=CC=	kaempfero	-9.58861
P55072	VCP	Valosin Co	NC(CO)C(	L-Serine	-5.30809
P55072	VCP	Valosin Co	OC1=CC2	Luteolin	-9.52808
P55072	VCP	Valosin Co	COC1=CC	Melatonin	-8.92806
P55072	VCP	Valosin Co	O[C@@H	Neochloro	-8.92061
P55072	VCP	Valosin Co	OC1=CC((	phlorogluc	-5.94142
P55072	VCP	Valosin Co	O=C1OC2	Psoralen	-8.76328
P55072	VCP	Valosin Co	OC1=CC2	Quercetin	-9.60809
P55072	VCP	Valosin Co	OC(=O)C1	Salicylic ac	-5.53242

P55072	VCP	Valosin Co	COC1=C((	Scopoletin	-8.1771
P55072	VCP	Valosin Co	CC1=CC=	Toluene	-5.53815
P55072	VCP	Valosin Co	C[N+]1=C	Trigonellin	-6.51537
P55072	VCP	Valosin Co	COC1=C2	Xanthotoxi	-8.2613
P55072	VCP	Valosin Co	[H]OC1=C	Xanthotox	-8.34505
