# Supplementary figures and images for: Bioinformatics and Deep Learning Approach to Discover Food-Derived Active Ingredients for Alzheimer’s Disease Therapy
Source: Foods. 2025 Jan 4;14(1):127. doi: 10.3390/foods14010127 (PMC11719994; doi:10.3390/foods14010127)

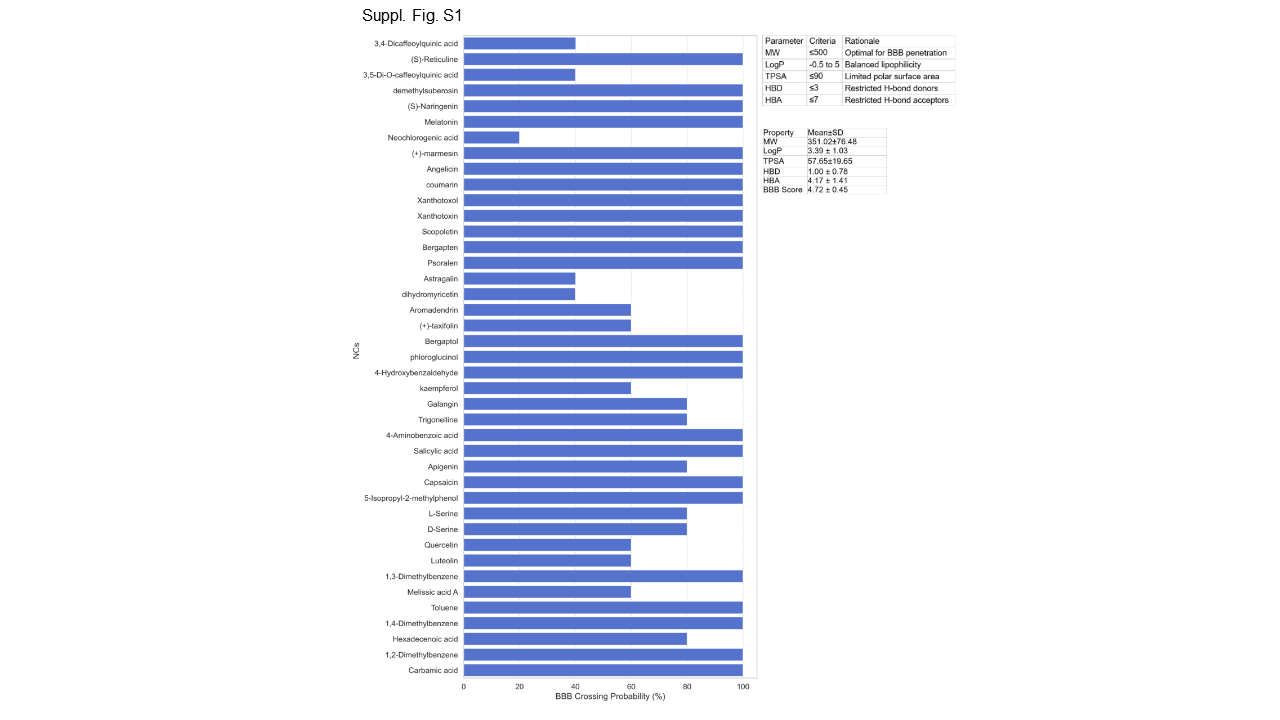

Supplement: Supplementary file 1 [file foods-14-00127-s001.zip › Suppl_Figure S1.tif]
